# The sterol-regulating human ARV1 binds cholesterol and phospholipids through its conserved ARV1 homology domain

**DOI:** 10.1016/j.jbc.2025.108306

**Published:** 2025-02-12

**Authors:** Jessie Lee Cunningham, Hsing-Yin Liu, Jamie Francisco, Karla.K. Frietze, J. Jose Corbalan, Joseph T. Nickels

**Affiliations:** 1 The Institute of Metabolic Disorders, Genesis Research and Development Institute, Hamilton, New Jersey, USA; 2 Rutgers Center for Lipid Research, New Jersey Institute for Food, Nutrition, and Health, Rutgers University, New Brunswick, New Jersey, USA

**Keywords:** cholesterol-binding protein, lipid, lipid transport, phosphoinositide, phospholipids, lipid-binding protein

## Abstract

Evidence suggests that ARV1 regulates sterol movement within the cell. *Saccharomyces cerevisiae* cells lacking ScArv1 have defects in sterol trafficking, distribution, and biosynthesis. HepG2 cells treated with *hARV1* antisense oligonucleotides accumulate cholesterol in the endoplasmic reticulum. Mice lacking Arv1 have a lean phenotype when fed a high fat diet and show no signs of liver triglyceride or cholesterol accumulation, suggesting a role for Arv1 in lipid transport. Here, we explored the direct lipid-binding activity of recombinant human ARV1 using *in vitro* lipid-binding assays. ARV1 lipid-binding activity was observed within the first N-terminal 98 amino acids containing the conserved ARV1 homology domain (AHD). The zinc-binding domain and conserved cysteine clusters within the AHD were necessary for lipid binding. Both full-length ARV1 and the AHD bound cholesterol, several phospholipids, and phosphoinositides with high affinity. The AHD showed the highest binding affinity for monophosphorylated phosphoinositides. Several conserved amino acids within the AHD were necessary for phospholipid binding. Biochemical studies suggested that ARV1 exists as a dimer in cells, with oligomerization being critical for ARV1 function, as amino acid mutations predicted to have a negative effect on dimerization caused weakened or complete loss of lipid binding. Our results show for the first time that human ARV1 can directly bind cholesterol and phospholipids. How this activity may function to regulate lipid binding and maintain proper lipid trafficking and/or transport in cells requires further studies.

Lipids make up the bilayers of all cell membranes (1). Various organelles are enriched in specific lipids, as cholesterol, sphingolipids, and PC^1^ accumulate in the plasma membrane (2), ceramides in the endoplasmic reticulum (ER) (3), and cardiolipin (CL) in the mitochondria (4). Lipid asymmetry occurs between the inner and outer membrane leaflets of organelles. The outer leaflet of the plasma membrane (PM) contains high levels of cholesterol, phosphatidylcholine (PC), and sphingomyelin, while phosphatidylinositol (PI), phosphatidylserine (PS), and phosphatidylethanolamine (PE) are found in the inner leaflet (5). Maintaining lipid asymmetry is a dynamic process with constitutive lipid exchange being the driving force. Intrinsic and extrinsic cell signals can temporally initiate lipid transfer as in the case of PS flipping from the inner to outer leaflet of the PM during apoptosis (6).

Unique lipid-specific transporter proteins (LTPs) regulate lipid bilayer composition and asymmetry, and their loss of function is associated with many metabolic disorders (7, 8, 9). LTP families are found in both prokaryotes and eukaryotes that show binding and transfer of single lipid classes (10, 11, 12). Transport is nonvesicular, carrier-mediated lipid transport (13). Examples include the ceramide transporter CERT (14) and the cholesterol-binding proteins, NPC1, NPC2, ABCA1, OSBPs, and ORPs (15, 16, 17, 18, 19, 20).

LTPs function in heterogeneous lipid flipping between organelles. One example is the yeast OSBP ortholog, Osh4, which transfers sterol from the ER to the Golgi in exchange for retrograde transport of phosphatidylinositol 4-phosphate (PI(4)P) (21). The ORPs perform similar functions at membrane contact sites (22). Sec14-like phosphatidylinositol transfer proteins are PI- and PC-binding proteins playing important roles in transporting PI from the ER to the PM for phosphoinositide (PIP) signaling (23). Tissue-specific fatty acid transfer proteins bind and transport fatty acids (24), while fatty acid binding proteins transport fatty acids, bile acids, and endocannabinoids (25). The super-family of LTPs that contain a repeating β-groove domain function in bulk lipid transport (26, 27).

Specific phospholipid transfer is carried out by lipid flippases, floppases, and scramblases. Flippases and floppases of the type-IV P-type ATPase class transfer PS and PE from the outer to inner leaflet of the PM, a process requiring ATP hydrolysis (28). Ca^2+^-dependent scramblases lack lipid substrate specificity, have bidirectional lipid transfer activity, and do not require ATP (29). Flippases possess a hydrophobic center cavity for lipid binding that allows for the exchange of lipids between membranes (30). Most flippases form heterodimers with CDC50 to ensure proper membrane localization (31).

*Saccharomyces cerevisiae* ScAre1 and ScAre2 are acyl-CoA:cholesterol acyltransferase yeast orthologs. *Scare1ΔScare2Δ* cells lack all sterol esterification activity yet are viable (32). *ScARV1* was identified in a synthetic lethal screen looking for genes making the *ScARE2* gene essential for *Scare1ΔScare2Δ* cell growth (33). Phenotypes of Sc*arv1Δ* cells include defects in sphingolipid and GPI anchor synthesis (34, 35), sterol trafficking and distribution (36, 37), ER membrane morphology (38), and fatty acid homeostasis (39). Lipid bilayer stress is induced in *Scarv1Δ* cells, leading to activation of the unfolded protein response and the cell wall integrity pathways (40, 41). *ScArv1Δ* cells are both sensitive to the ergosterol-binding antifungal agent, nystatin, and are unable to take up ergosterol, suggesting defects in ergosterol uptake and trafficking to the PM (33).

Knockdown of human *ARV1* in HepG2 cells causes the accumulation of cholesterol in the ER (41). *Arv1*^*−/−*^ mice fed a high fat diet have a lean phenotype, lower levels of triglycerides and cholesterol in their livers, and lack any signs of MASLD/MASH, suggesting a critical role for Arv1 in regulating lipid transport and deposition in higher eukaryotes (42, 43).

Human ARV1 is a 271 amino acid protein that is predicted to contain between 3 to 5 transmembrane domains depending on the analysis program used (33). It contains a highly conserved ARV1 homology domain (AHD) at its N-terminus that has a zinc-binding motif containing two conserved cysteine clusters. Proteomic studies have shown that human ARV1 may bind cholesterol and fatty acid bioactive probes (44, 45). These studies were performed in cells, however, so whether ARV1 directly binds lipids remains unanswered.

Here we performed *in vitro* lipid-binding studies using recombinant human ARV1. Our results show that ARV1 binds several lipid species with varying affinities that include several phospholipids, PIPs, and cholesterol. The AHD domain and zinc-binding motif are necessary for lipid binding. Finally, our results strongly suggest that ARV1 binds lipids as a dimer in cells.

## Results

### The AHD cosediments with cholesterol-containing liposomes

**Figure 1:**
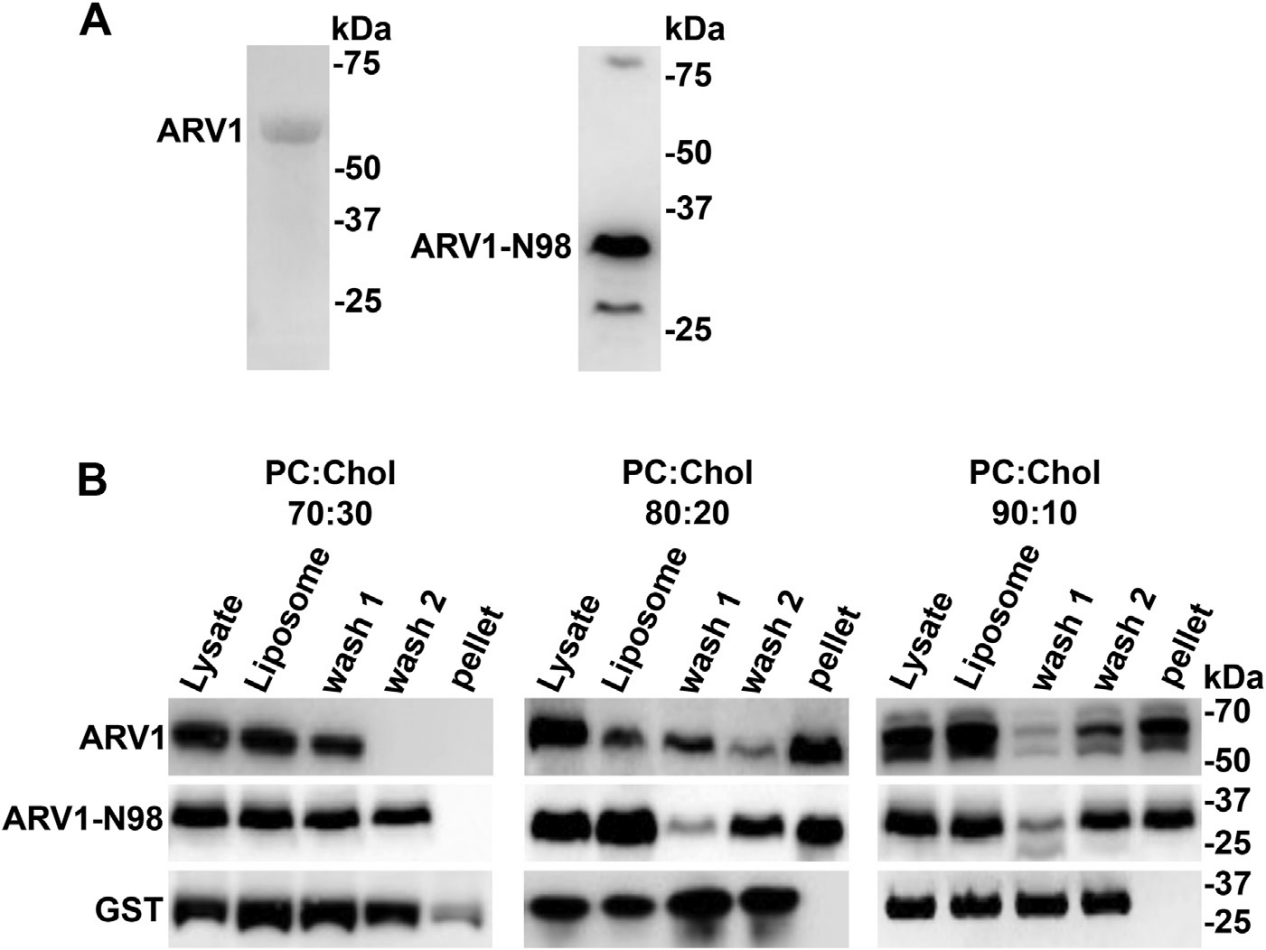
**Full-length ARV1 and N98 associate with cholesterol-containing liposomes.** Phosphatidylcholine:cholesterol liposomes were generated as described in the “Experimental procedures” section. An aliquot of purified GST-tagged protein (lysate) was mixed with liposomes (liposome) and spun at 50,000*g* for 20 min. An aliquot of the supernatant was taken (wash 1) and the pellet was washed a second time. An aliquot of the second supernatant was taken (wash 2) and the pellet was resuspended (pellet). All washes and resuspensions of pellets were performed using floatation buffer. Proteins were resolved by SDS-PAGE and detected by Western blotting. *A*, Western blot of GST-ARV1 and GST-N98. *B*, Western blots of GST-ARV1 and GST-ARV1-N98 tagged protein binding to different concentration PC:Chol liposomes. Blots are representative images. (n = 5).

The loss of ARV1, whether through gene knockdown (46) or ablation (42, 43), results in defects in sterol transport. Previous work has shown that ARV1 bound a cholesterol bioactive probe in cells, but whether this was a direct lipid–protein interaction could not be resolved (44, 45). We used glutathione Sepharose chromatography to purify *Escherichia coli*–expressed N-terminal GST-tagged full-length GST-ARV1 (ARV1) and a GST-N98 (ARV1-N98) protein that contained the AHD and tested for their binding to cholesterol using PC:Chol liposomes made up of increasing percentages of cholesterol (10–70%) (Fig. 1*A* and S1).

While neither ARV1 nor ARV1-N98 associated with PC:Chol liposomes containing 30 to 50% cholesterol, both bound to PC liposomes containing 10% and 20% cholesterol (Fig. 1*B*). GST weakly bound to PC:Chol liposomes containing 30% cholesterol.

### ARV1 and ARV1-N98 interact with phospholipids

There are many examples of lipid transporters binding more than one lipid species (6, 47, 48). Thus, we tested whether ARV1 and/or ARV1-N98 could interact with any glycerophospholipids, neutral lipids, sphingolipids, and/or PIPs by performing far westerns using lipid-embedded membrane strips. GST-AnnexinA2 (Annexin A2), a known phospholipid-binding protein, was used as a positive control for protein–lipid interaction (49, 50). GST protein–lipid interactions were used as controls for nonspecificity.

**Figure 2:**
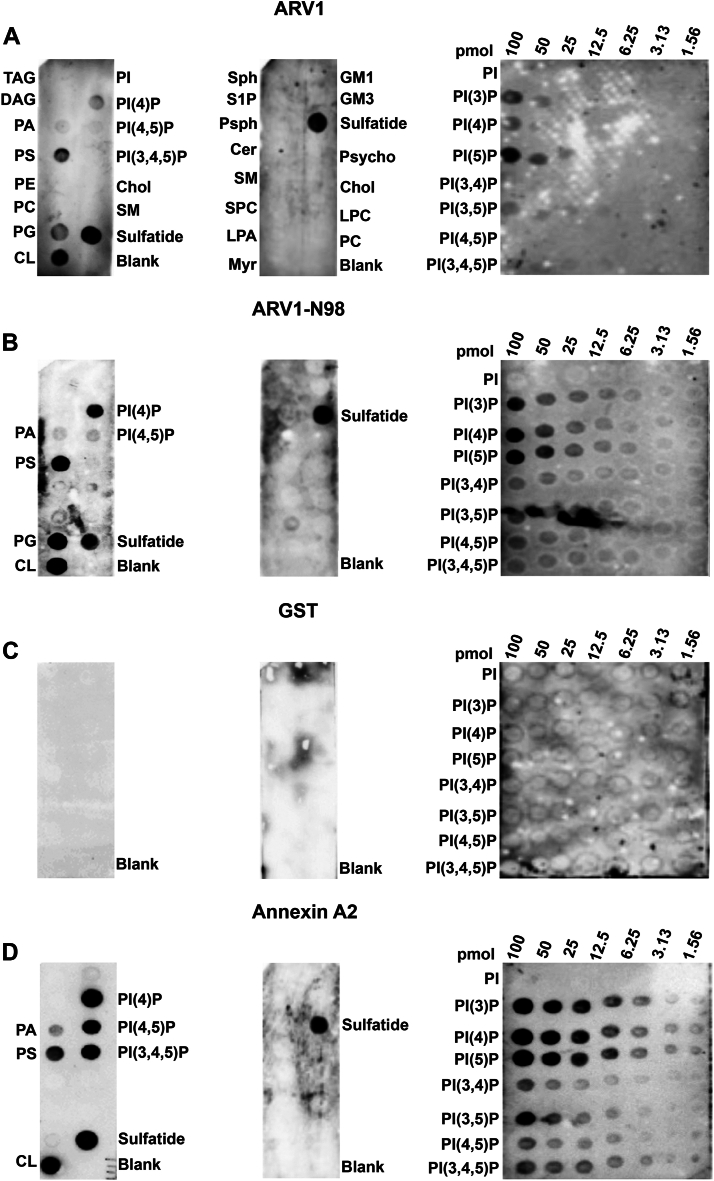
**Full-length ARV1 and ARV1-N98 interact with phospholipids and phosphoinositides.** Lipid overlay far westerns were performed using GST-ARV1, GST-ARV1-N98, GST, and GST-Annexin A2. *A*, GST-ARV1 lipid overlay interactions. *B*, GST-ARV1-N98 lipid overlay interactions. *C*, GST lipid overlay interactions. *D*, GST-Annexin A2 lipid overlay interactions. Blots are representative images. (n = 5).

ARV1 interacted with PS, CL, sulfatide, and weakly to phosphatidylglycerol (PG) and PI(4)P (Fig. 2*A*). ARV1-N98 interacted with PS, PI(4)P, PG, CL, and sulfatide (Fig. 2*B*). GST showed no cross-reactivity with any lipid species (Fig. 2*C*). Annexin A2 interacted with PS, PI(4)P, PI(4,5)P_2_, PI(3,4,5)P_3_, CL, sulfatide, and weakly to phosphatidic acid (PA) (Fig. 2*D*).

We next used a sphingolipid membrane strip, which showed ARV1 and ARV1-N98 again interacted with sulfatide, but not with sphingosine, sphingosine-1-phosphate, phytosphingosine, the gangliosides, GM1 and GD3, or ceramide (Fig. 2, *A* and *B*). Interactions were also not seen for cholesterol, PE, lyso-phosphatidic acid, and lyso-phosphatidylcholine (Fig. 2, *A* and *B*). GST did not cross react with any lipid species (Fig. 2*C*), while Annexin A2 also interacted with sulfatide (Fig. 2*D*).

To define PIP specificity, we used a PIP lipid membrane strip containing decreasing concentrations of lipid per spot (100pmol-1.56pmol). ARV1 interacted with phosphatidylinositol 3-phosphate (PI(3)P) and PI(5)P and weakly to PI(4)P (Fig. 2*A*). ARV1-N98 interacted with PI(3)P, PI(4)P, and PI(5)P (Fig. 2*B*). GST showed no cross-reactivity (Fig. 2*C*). Annexin A2 showed the strongest interactions with PI(3)P, P(4)P, and PI(5)P, followed by phosphatidylinositol 3,5-bisphosphate (PI(3,5)P_2_) and PI(3,4,5)P_3_ (Fig. 2*D*).

### ARV1-N98 protein lipid interaction sites are found within zinc-binding motif

**Figure 3:**
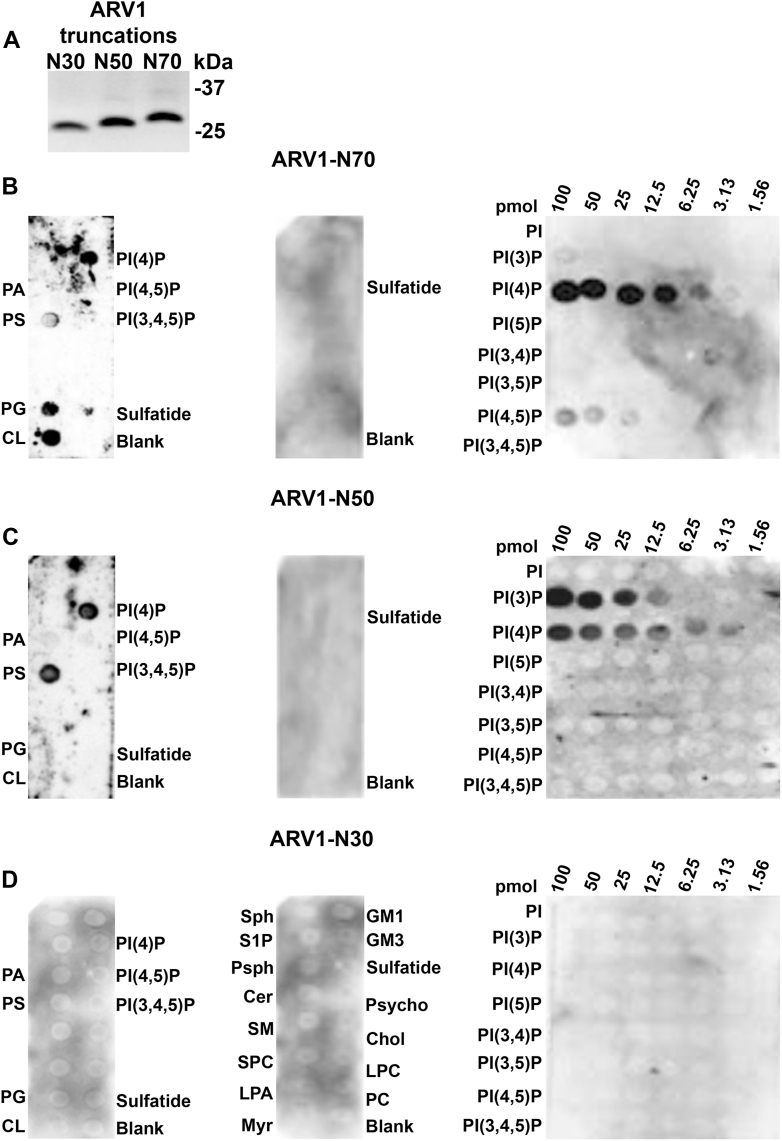
**The zinc-binding motif within the AHD is required for lipid–protein interaction.***A*, carboxy-terminal GST-ARV1 truncations were generated and used in lipid overlay far westerns. *B*, GST-ARV1-N70 lipid-binding interaction. *C*, GST-ARV1-N50 lipid-binding interaction. *D*, GST-ARV1-N30 lipid-binding interaction. Blots are representative images. (n = 5).

So far, our results suggested that ARV1 and ARV1-N98 shared the same lipid-binding profile. ARV1-N98 harbors a zinc-binding motif that resides between amino acids 34 to 61. To further define lipid-binding motifs within ARV1, we made sequential carboxy-terminal amino acid truncations of ARV1-N98 that included deleting some or all the zinc-binding motif (Figs. 3*A* and S1).

Upon deleting 28 carboxy-terminal amino acids of ARV1-N98 (ARV1-N70), we observed a loss of interaction with sulfatide and reduced interactions with PS, PI(3)P, and PI(5)P, but enhanced PI(4)P binding (Figs. 3*B* *versus* 2*B*). Eliminating an additional 20 amino acids (ARV1-N50) eliminated the PG, CL, and PI(5)P interactions (Figs. 3*B* *versus* 2*B*) but enhanced the PI(3)P interaction and restored the PS interaction (Fig. 3*B* *versus C*). A GST fusion protein containing the first 30 N-terminal amino acids (ARV1-N30) of ARV1, which lacks the entire AHD and zinc-binding motif, lost all ability to interact with any lipid (Fig. 3*D*). These results indicate that the AHD and zinc-binding motif are important for all ARV1 protein–lipid interactions.

### Several conserved amino acids within the ARV1-N98 protein define phospholipid interaction preference

**Figure 4:**
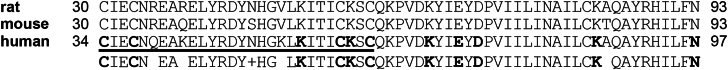
**Amino acids comprising the human, rat, and mouse AHD.** The zinc-binding domain is underlined. Identical amino acids are below. Conserved amino acids in bold were mutated.

**Figure 5:**
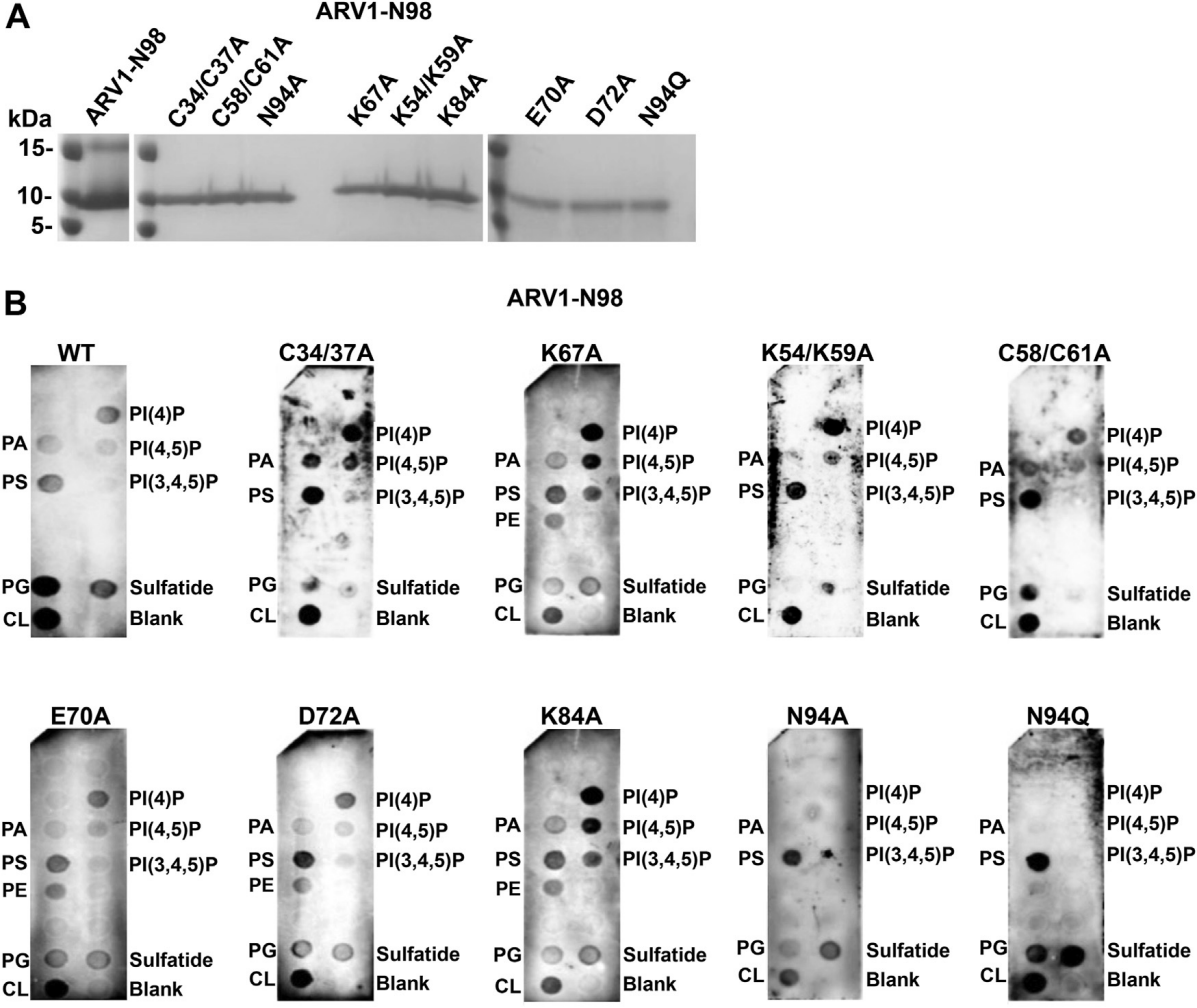
**Several conserved amino acids within the zinc-binding domain are required for specific lipid–protein interactions.** Far westerns were performed using specific 6XHIS-ARV1-N98 mutant proteins (*A*). All amino acids residues were changed to alanine unless otherwise indicated. *B*, various site-directed mutants far westerns. Blots are representative images. (n = 5).

We next decided to map out the amino acids within the AHD required for various phospholipid (PL) interactions by performing site-directed mutagenesis on conserved amino acids within a 6XHIS-ARV1-N98 fusion protein (Fig. 4; bold) (Figs. 5*A* and S1).

We found that mutating the cysteines together within each cluster alone to alanines (C34/C37A; C58/C61A) eliminated any interaction with sulfatide and weakened or eliminated the PG interaction but enhanced the PS interaction compared to the ARV1-N98 protein (Fig. 5*B*). The C34/C37A double mutant also showed enhanced interactions with PI(4)P and PI(4,5)P_2_. Mutating K54/K59 to alanines abolished PG and sulfatide interactions while increasing the interaction with PI(4)P. Mutating K67 to alanine weakened the PG and sulfatide interactions but increased interactions with (PI(4)P, PI(4,5)P_2_, and PI(3,4,5)P_3_. Mutating K84 to alanine also increased interactions with PI(4)P, PI(4,5)P_2_, and PI(3,4,5)P_3_, while alanine mutations at E70, D72, and K84 weakened PG and CL interactions but enhanced interaction with PE (Fig. 5*B*). Mutating Asn94 to an alanine (N94A) completely abolished interactions with all PIP species, as did mutating this residue to glutamate (N94Q), but the glutamate mutant now interacted more strongly with PG, CL, and sulfatide (Fig. 5*B*). The K67A, E70A, D72A, and K84A mutants interacted weakly with PE. These results indicate the presence of multiple lipid binding and/or regulatory domains within the AHD.

### ARV1 and ARV1-N98 show PC:PL liposome-binding preference

To further explore ARV1 lipid-binding interactions, we performed liposome-binding assays. PC:PL liposomes represent a more physiologically relevant membrane environment to test lipid-protein binding (51, 52). Proteins show little binding to PC, so liposomes made up of PC act as carriers for various specific lipids to be tested for binding affinity. We generated PC:PL mixed liposomes and used ultracentrifugation to determine the PL binding preferences of GST-ARV1 and GST-ARV1-N98. Liposomes that were incubated with fusion protein were ultracentrifuged and pelleted twice, with washings in between, and lipid-bound proteins were detected from the final pellet by SDS-PAGE and western analysis.

**Figure 6:**
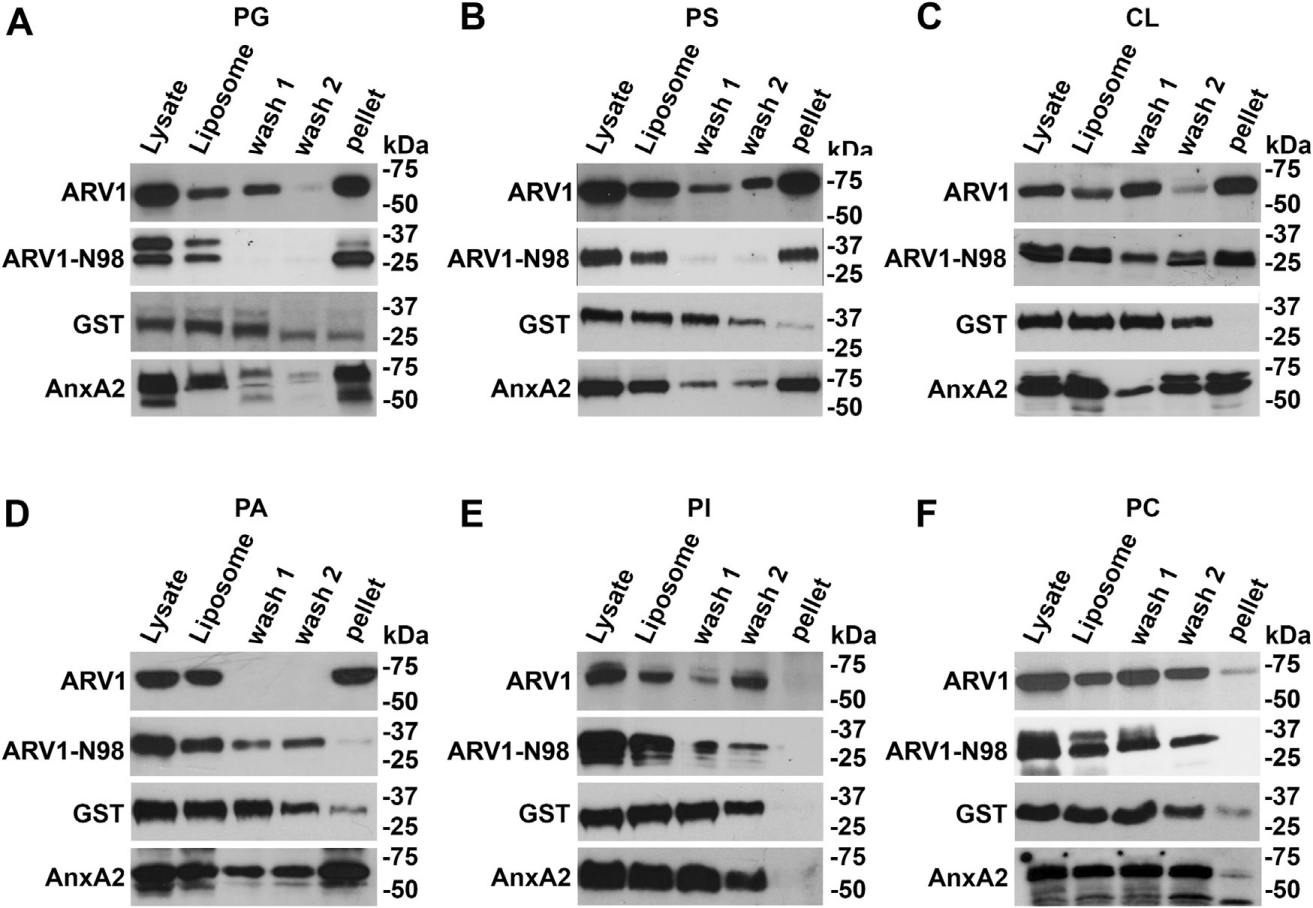
**Full-length ARV1 and N98 proteins associate with phospholipid liposomes.** Liposomes were made as described in figure legend 1. GST-ARV1, GST-ARV1-N98, GST, and GST-Annexin A2 were used for all experiments. *A*, protein binding to PG liposomes. *B*, protein binding to PS liposomes. *C*, protein binding to CL liposomes. *D*, protein binding to PA liposomes. *E*, protein binding to PI liposomes. *F*, protein binding to PC liposomes. GST was used as a control for nonspecific liposome association. Blots are representative images. (n = 5).

We found that ARV1 bound to PG, PS, CL, and PA liposomes (Fig. 6, *A*–*D*) but not with PI liposomes and very weakly with PC liposomes (Fig. 6, *E* and *F*). ARV1-N98 bound to PG, PS, and CL liposomes (Fig. 6, *A*–*C*) but not PA or PI liposomes (Fig. 6, *D*–*F*). GST protein did show a very weak binding to PG, PA, and PC liposomes (Fig. 5, *A*, *D* and *F*). AnnexinA2 bound to PG, CL, PS, and PA liposomes and very weakly to PC liposomes (Fig. 6, *A*–*D*, *F*) but not to PI liposomes (Fig. 6*E*). Neither ARV1, ARV1-N98, GST, nor AnnexinA2 bound to PE liposomes.

### The conserved cysteine clusters within the zinc-binding domain are required for specific ARV1-N98 PC:PL liposome binding

**Figure 7:**
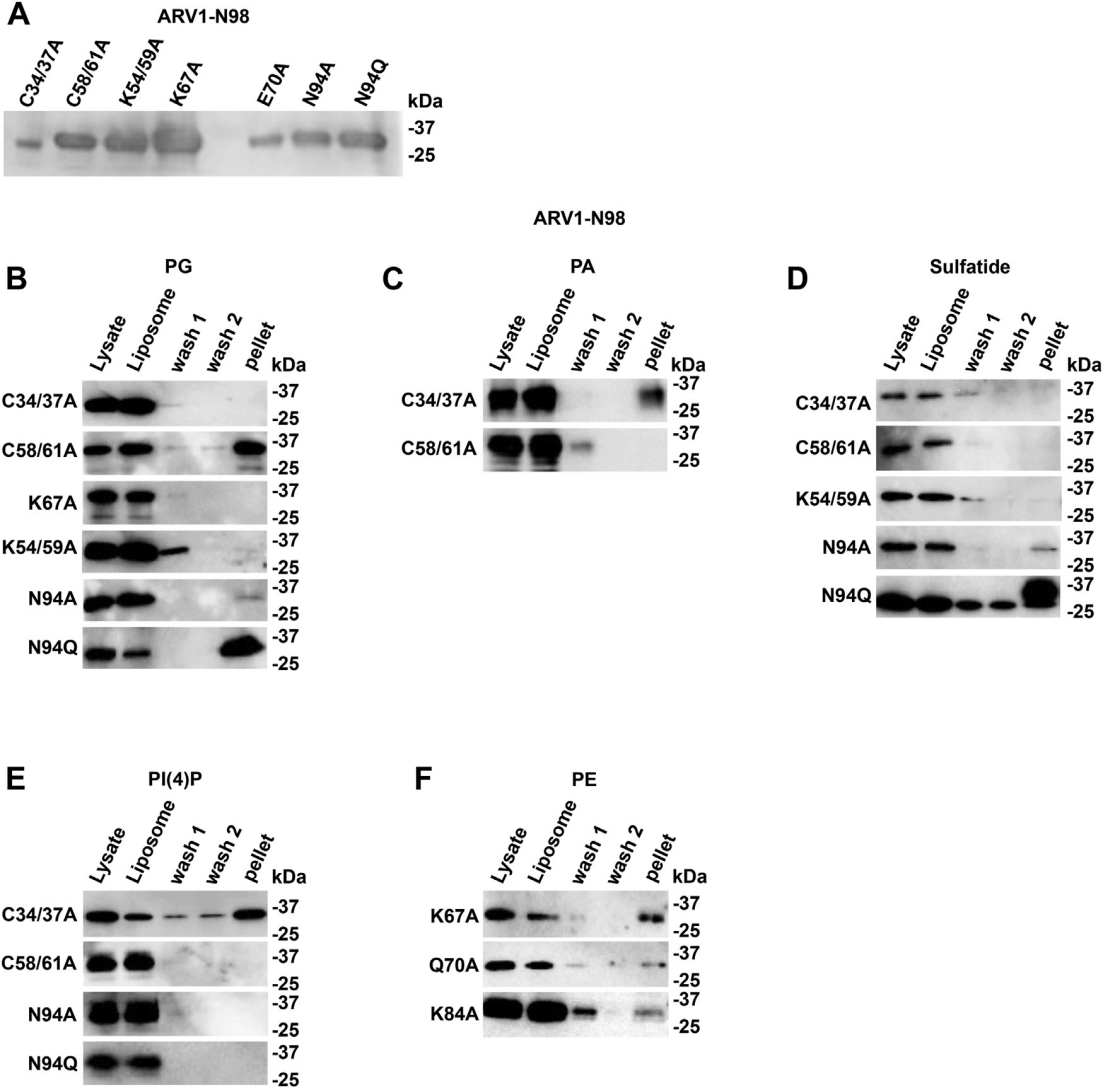
**The Cys58/Cys61 cluster within the zinc-binding domain is required for N98 association with specific phospholipid-containing liposomes.***A*, GST-ARV1-N98 mutants were tested for liposome association. *B*, PG liposome binding. *C*, PA liposome binding. *D*, sulfatide-binding association. *E*, PI(4)P liposome binding. *F*, PE liposome binding. Blots are representative images. (n = 5).

Specific 6XHIS3-ARV1-N98 site-directed mutants showing reduced PL interactions in our lipid overlay assays were expressed as GST fusion proteins and tested for association with various PC:PL liposomes (Fig. 7*A*).

We found that the C34/C37A mutant bound PA and PI(4)P liposomes but not PG or sulfatide liposomes (Fig. 7, *B*–*E*). Moreover, the C58/C61A mutant did not bind PA, sulfatide, or PI(4)P liposomes (Fig. 7, *C*–*E*), while the K54/K59A and K67A mutants were unable to bind PG or sulfatide liposomes (Fig. 7, *B* and *D*), and the N94A and N94Q mutants lost the ability to bind PI(4)P liposomes (Fig. 7*E*). The N94A mutant showed very weak PG and sulfatide liposome binding (Fig. 7, *B* and *D*). Interestingly, the K67A, E70A, and K84A mutants showed binding to PE liposomes (Fig. 7*F*), like what we observed in our lipid overlay assays. Overall, our liposome results directly correlated with the lipid overlay far western results and further substantiate the hypothesis that multiple binding sites and/or lipid regulatory domains reside within the AHD.

### The ARV1-N98 protein show differential binding affinities for glycerophospholipids and PIPs

We used homogeneous time resolved fluorescence (HTRF) assays with recombinant 6XHIS-ARV1-N98 and biotinylated-phospholipids and biotinylated-PIPs to determine phospholipid-binding affinity and obtain half maximal effective concentration values (EC_50_) (53, 54, 55).

**Figure 8:**
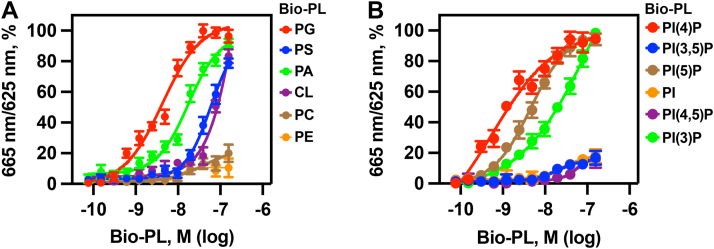
**N98 binds phospholipids with different affinities.** HTRF assays were performed using 6XHIS-ARV1-N98 proteins. A constant concentration of 6XHIS-ARV1-N98 (0.75 μM) was used in the presence of increasing concentrations of biotinylated phospholipids. EC_50_ values were obtained using GraphPad Prism statistical analysis. *A*, 6XHIS-ARV1-N98 binding to biotinylated phospholipids. *B*, 6XHIS-N98 binding to biotinylated phosphoinositides.

We found that ARV1-N98 had the highest binding affinity for Bio-PG with an EC_50_ of 4.3 × 10^−9^ M (Fig. 8*A*, red circles), followed by Bio-PA with an EC_50_ of 1.6 × 10^−8^ M (Fig. 8*A*, green circles), Bio-PS (Fig. 8*A*, blue circles; EC_50_ values of 3.4 × 10^−7^ M), and Bio-CL (brown circles; 4.7 × 10^−7^ M) (Table 1). Very weak if any binding was detected to Bio-PE (Fig. 8*A*, orange circles; 5.3 × 10^−6^ M) and Bio-PC (Fig. 8*A*, purple circles; 1.1 × 10^−6^ M).

**Table 1:** EC_50_ values for 6XHIS-N98

Bio-PL	EC_50_, M ± S.D.
PG	4.3 × 10^−9^ ± 2.1 × 10^−10^
PS	3.4 × 10^−7^ ± 1.1 × 10^−8^
PA	1.6 × 10^−8^ ± 1.2 × 10^−9^
CL	4.7 × 10^−7^ ± 1.8 × 10^−8^
PC	1.1 × 10^−6^ ± 1.6 × 10^−7^
PE	5.3 × 10^−6^ ± 1.3 × 10^−7^
PI	1.2 × 10^−5^ ± 7.8 × 10^−6^
PI(3)P	1.5 × 10^−8^ + 1.3 × 10^−9^
PI(4)P	4.7 × 10^−11^ + 4.1 × 10^−12^
PI(5)P	1.6 × 10^−9^ + 2.2 × 10^−10^
PI(3,5)P	2.2 × 10^−6^ + 1.7 × 10^−6^
PI(4,5)P	6.7 × 10^−6^ + 1.4 × 10^−6^

The ARV1-N98 protein bound all three mono-phosphorylated PIPs, with the highest binding observed for PI(4)P (Fig. 8*B*, red circles; 4.7 × 10^−11^ M), followed by PI(5)P (Fig. 8*B*, brown circles; 1.6 × 10^−9^ M) (brown circles) and PI(3)P (Fig. 8*B*, green circles; 1.54 × 10^−8^ M). ARV1-N98 showed very little if any binding to PI (Fig. 8*B*, orange circles; 1.2 × 10^−5^ M), PI(3,5)P_2_ (Fig. 8*B*, blue circles; 2.2 × 10^−6^ M), and PI(4,5)P_2_ (Fig. 8*B*, purple circles; 6.7 × 10^−6^ M).

### Mutations within the zinc-binding motif and cysteine clusters result in altered phospholipid-binding affinity

Several conserved amino acids were critical for ARV1-N98 interacting with specific phospholipids. Thus, we once again used HTRF and tested these mutants for lipid binding to obtain EC_50_ values using the 6HIS-ARV1-N98 protein.

We found that the K54/K59A, K67A, K84A, and N94A mutants showed reduced PG-binding affinities (Table 2). The C34/37A also showed reduced affinity for PG but increased PA-binding affinity. Several other mutants showed reduced binding to PA (K54/K59A, C58/C61A, E70A, D72A, N94A, and N94Q), with a subset of these mutants also harboring weakened PI(4)P-binding affinity (C58/C61A, K67A, E70A, D72A, N94A, and N94Q).

**Table 2:** EC_50_ values for 6XHIS-N98 site-directed mutants

	Bio-PL, EC_50_, M ± S.D.					
	PG	PS	PA	CL	PC	PE
N98	4.3 × 10^−9^ ± 2.1 × 10^−10^	3.4 × 10^−8^ ± 1.1 × 10^−9^	1.6 × 10^−8^ ± 1.2 × 10^−9^	4.7 × 10^−7^ ± 1.8 × 10^−8^	1.1 × 10^−6^ ± 1.6 × 10^−7^	5.3 × 10^−6^ ± 1.3 × 10^−7^
C34/37A	3.2 × 10^−7^ ± 1.2 × 10^−8^	NC	8.3 × 10^−9^ ± 6.7 × 10^−10^	NC	NC	NC
C58/61A	NC	NC	9.6 × 10^−6^ ± 7.3 × 10^−8^	NC	NC	NC
K54/K59A	9.1 × 10^−6^ ± 1.3 × 10^−8^	NC	2.9 × 10^−6^ ± 2.1 × 10^−7^	NC	NC	NC
K67A	7.4 × 10^−6^ ± 6.2 × 10^−7^	NC	NC	5.9 × 10^−7^ ± 3.2 × 10^−8^	NC	3.5 × 10–7 ± 4.2 × 10^−8^
E70A	NC	NC	8.4 × 10^−6^ ± 4.2 × 10^−8^	NC	NC	1.9 × 10^−7^ ± 1.3 × 10^−8^
D72A	NC	NC	5.7 × 10^−6^ ± 5.1 × 10^−8^	NC	NC	NC
K84A	7.6 × 10^−6^ ± 4.1 × 10^−8^	NC	NC	1.1 × 10^−7^ ± 5.1 × 10^−8^	NC	2.1 × 10^−7^ ± 4.3 × 10^−8^
N94A	5.8 × 10^−7^ ± 1.7 × 10^−8^	NC	6.9 × 10^−6^ ± 7.1 × 10^−8^	NC	NC	NC
N94Q	NC	NC	7.2 × 10^−6^ ± 2.2 × 10^−8^	NC	NC	NC

NC, no change.

### ARV1-N98 lipid binding is differentially altered by the presence of specific phospholipids

Liposome interaction studies are commonly used to determine how protein–lipid interactions are influenced through the addition of a “competing” lipid(s). Studies like this have been used to obtain kinetic constants for several phospholipid enzymes in yeast (56, 57), PKC (58, 59), and PLA_2_ (60).

HTRF screening assays have been used to identify inhibitors of the CERT–ceramide interaction (61), FXR agonists (62), and PGE_2_ inhibitors (63), where IC_50_ values were determined for inhibitor compounds. Thus, we used HTRF assays to determine IC_50_ values for individual PLs that were acting as competitor lipids.

Competition assays were performed using HTRF with a constant concentration of 6XHIS-ARV1-N98 protein and a constant concentration of biotinylated phospholipid. Biotinylated phospholipid concentration was set at a concentration that gave an affinity signal that was 80% that of the maximum signal detected. Increasing concentrations of the nonbiotinylated “competitor” lipid was sequentially added at 1:2 serial dilutions beginning at 25x the concentration of the biotinylated phospholipid.

**Figure 9:**
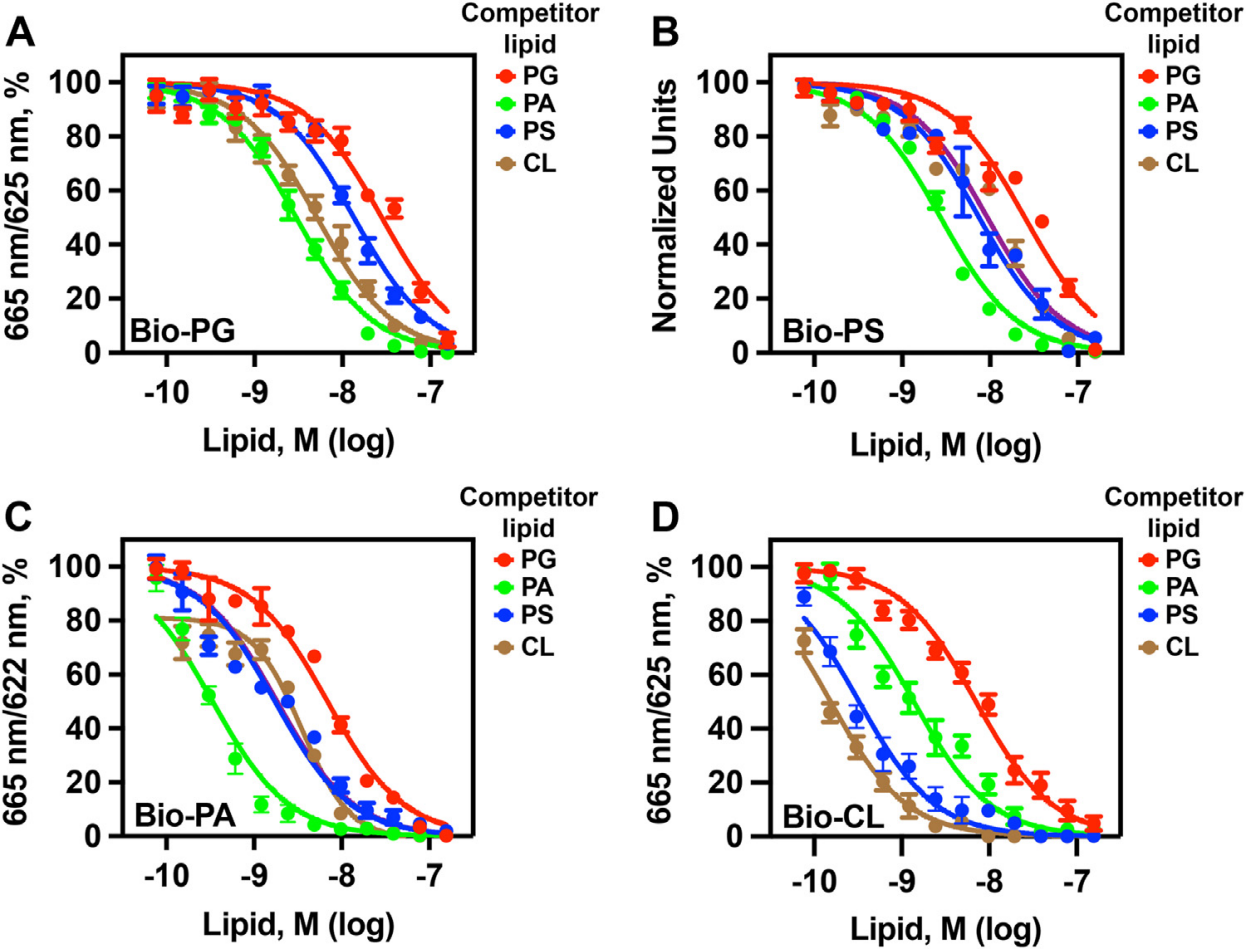
**Various phospholipids affect ARV1-lipid binding.** For competition assays, 6XHIS-ARV1-N98 protein was held at a single concentration (0.75 μM) and a single biotinylated phospholipid was held at a constant concentration (80% maximum signal). Increasing concentrations of nonbiotinylated competitor lipids were added and IC_50_ values were obtained using GraphPad Prism statistical analysis. *A*, biotinylated-PG in the presence of the indicated nonbiotinylated PLs. *B*, biotinylated-PS in the presence of the indicated nonbiotinylated PLs. *C*, biotinylated-PA in the presence of the indicated nonbiotinylated PLs. *D*, biotinylated-CL in the presence of the indicated nonbiotinylated PLs. Statistical analysis is described in “Experimental procedures.”

We found that ARV1-N98 binding to Bio-PG was reduced by increasing concentrations of PA > CL > PS > PG (Fig. 9*A*). ARV1-N98 binding to Bio-PS was also reduced (PA > PS=CL > PG) (Fig. 9*B*). Similar results were seen for Bio-PA (PA > PS=CL > PG) (Fig. 9*C*) and Bio-CL (CL > PS > PA > PG) (Fig. 9*D*). IC_50_ values are listed in Table 3.

**Table 3:** IC_50_ values for competitor lipids

Lipid	Kinetic constant values
	EC50, M ± S.D.
Bio-PG	4.3 × 10^−9^ ± 2.1 × 10^−10^
Competitor lipid	IC50, M ± S.D.
PG	2.8 × 10^−8^ ± 1.6 × 10^−9^
PS	1.3 × 10^−8^ ± 1.7 × 10^−9^
PA	3.1 × 10^−9^ ± 1.5 × 10^−10^
CL	5.1 × 10^−9^ ± 2.3 × 10^−10^
	EC50, M ± S.D.
Bio-PS	3.4 × 10–8 ± 1.1 × 10–9
Competitor lipid	IC50, M + S.D.
PG	2.5 × 10^−8^ ± 8.1 × 10^−10^
PS	7.5 × 10^−9^ ± 3.7 × 10^−10^
PA	2.7 × 10^−9^ ± 3.1 × 10^−10^
CL	9.3 × 10–9 ± 5.6 × 10^−10^
	EC50, M ± S.D.
Bio-PA	1.6 × 10^−8^ ± 1.2 × 10^−9^
Competitor lipid	IC50, M ± S.D.
PG	6.9 × 10^−9^ ± 1.8 × 10^−10^
PS	1.7 × 10^−9^ ± 2.2 × 10^−10^
PA	3.3 × 10^−10^ ± 3.9 × 10^−11^
CL	1.8 × 10^−9^ ± 6.2 × 10^−10^
	EC50, M ± S.D.
Bio-CL	4.7 × 10^−7^ ± 1.8 × 10^−8^
Competitor lipid	IC50, M ± S.D.
PG	7.1 × 10^−9^ ± 2.6 × 10^−10^
PS	3.2 × 10^−10^ ± 2.2 × 10^−11^
PA	1.4 × 10^−9^ ± 3.9 × 10^−10^
CL	1.5 × 10^−10^ ± 6.2 × 10^−11^

### ARV1 forms a dimer in cells

The human ARV1 alphaFold monomeric structure shows no homology to any lipid transporter or lipid-binding protein in eukaryotes or prokaryotes. Moreover, there are no human homologs within the genome that could give insight into ARV1 function. The P4A lipid flippases act as heterodimers to translocate lipids, associating with CDC50 (64). The lipid scramblase Atg9 that is involved in autophagy is a homotrimer (65). Our modeling predictions suggested that ARV1 could exist as a dimer, trimer, or hexamer (*J. Corbalan et al., in press*) this has been published PMID # 39845563. Thus, we determined if ARV1 could form a multimeric structure and if so, was oligomerization important for lipid binding.

**Figure 10:**
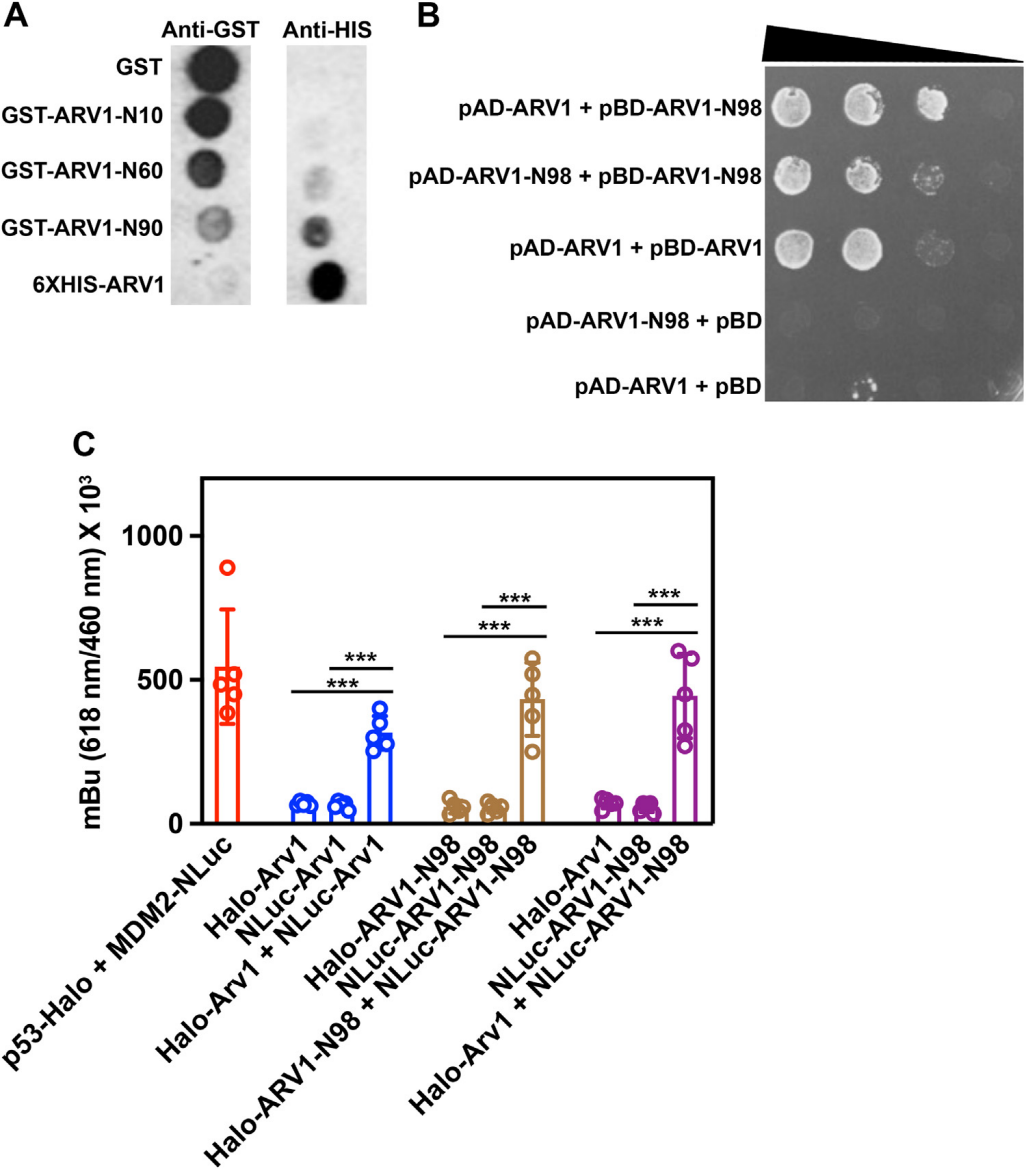
**ARV1 may form oligomers.***A*, nitrocellulose blots embedded with the indicated proteins were incubated with 6XHIS-ARV1 protein and subsequently probed with GST- or 6XHIS-antibodies. *B*, serial dilutions of yeast strains harboring various combinations of yeast two-hybrid ARV1/ARV1-N98 expression plasmids were grown on protein–protein interaction medium for 7 days. *C*, cells expressing various ARV1/ARV1-N98 NanoBRET plasmids were assayed for protein–protein interaction as described in “Experimental procedures.” Values are mean ± S.D. ∗∗∗*p* ≤ 0.0001. Blot is a representative image. (n = 5). Two way ANOVA was used for statistical analysis.

We first used far western analysis to probe for interactions between full-length 6XHIS-ARV1 and several GST-ARV1-N98 truncations. GST, GST-ARV1-N90, GST-ARV1-N60, GST-ARV1-N10, and 6XHIS-ARV1 were dotted onto nitrocellulose membranes and incubated with 6XHIS-ARV1 protein. GST and all GST fusion proteins were detected by anti-GST antibodies, while 6XHIS-ARV1 was not detected (Fig. 10*A*). Probing with anti-6XHIS antibodies detected the 6XHIS-ARV1 protein and the GST-ARV1-N90 protein (Fig. 10*A*). No binding was detected between 6XHIS-ARV1 and GST-ARV1-N60, GST-ARV1-N10, or GST.

Next, we used the yeast two-hybrid assay to test for any protein–protein interactions using combinations of full-length ARV1 and ARV1-N98. We found that yeast strains expressing the negative control vector combinations pAD-ARV1 + pBD or pAD-ARV1-N98 + pBD were unable to grow on amino acid drop out medium (leu^-^ trp^-^ his^-^) that selects for protein–protein interactions (Fig. 10*B*). Yeast strains expressing the full-length ARV1 (pAD-ARV1 + pBD-ARV1), ARV1-N98 (pAD-ARV1-N98 + pBD-ARV1-N98), and the full-length ARV1 and ARV1-N98 combination (pAD-ARV1 + pBD-ARV1-N98) all grew on the interaction selective medium (Fig. 10*B*). These same interactions were seen in HEK293 cells expressing full-length and ARV1-N98 Nano-Luc or Halo-tag fusions using a NanoBRET cell-based assay, which detects protein–protein interactions (Fig. 10*C*).

**Figure 11:**
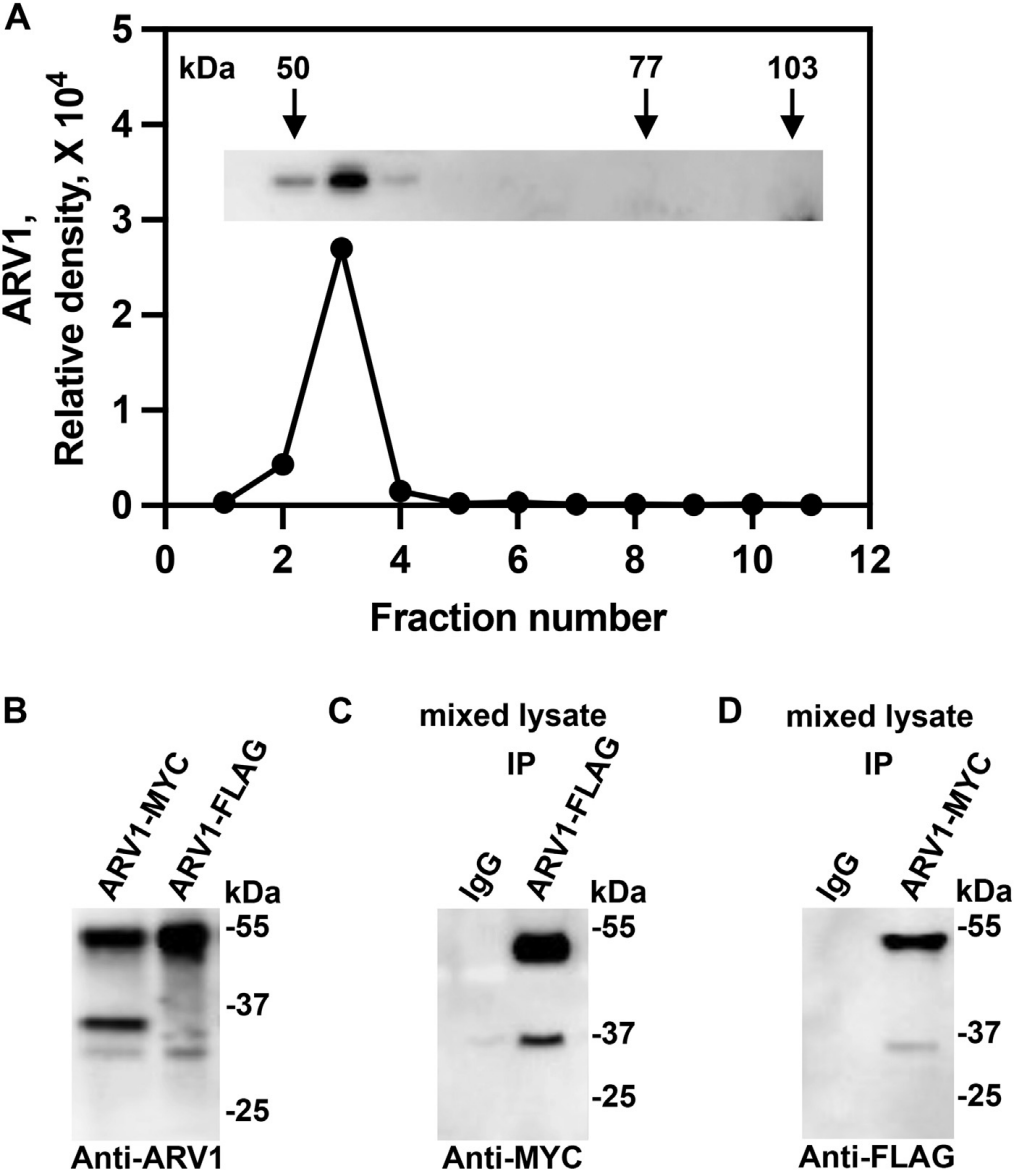
**ARV1 may form a dimer in cells.***A*, *Escherichiacoli* expressed purified full-length ARV1 was run on a 2.5%-25% sucrose density gradient. Gradient fractions were resolved by SDS-PAGE and western analysis was performed using anti-ARV1 antibodies (inset). Molecular weight markers from *left* to *right* are ovalbumin (50 kDa), bovine serum albumin (77 kDa), and phosphorylase β (103 kDa). *B*, Western blot of *E. coli* lysates expressing ARV1-MYC or ARV1-FLG probed with anti-ARV1 antibodies. *C*, mixed lysates were immunoprecipitated with anti-FLAG antibodies; co-immunoprecipitated proteins were resolved by SDS-PAGE and transferred to nitrocellulose and probed with anti-MYC antibodies. *D*, mixed lysates were immunoprecipitated with anti-MYC antibodies; co-immunoprecipitated proteins were resolved by SDS-PAGE and transferred to nitrocellulose and probed with anti-FLAG antibodies. IgG antibodies were used as a control for nonspecific binding. Blots are representative images. (n = 5).

We next ran purified ARV1 through a sucrose density gradient to determine its native molecular weight based on comparison to the density of various individual markers. The ARV1 protein was found in the fraction right after the 50 kDa and before the 77 kDa molecular weight markers, suggesting dimer formation (Fig. 11*A*).

We next expressed ARV1-MYC and ARV1-FLAG proteins from *E*. *coli* and performed lysate mixing/co-immunoprecipitation experiments (Fig. 11*B*). In this experiment, each tagged protein was expressed separately, and individual cell lysates were mixed. The lysate was then immunoprecipitated using an antibody against one tag and then a western analysis was performed on co-immunoprecipitates using an antibody against the other tag. If ARV1 oligomerizes in cells, an ARV1-MYC-ARV1-FLAG multimeric protein should be pulled down. Each antibody alone should pull down the oligomer. A Western blot using an anti-MYC antibody should detect the presence of the anti-FLAG immunoprecipitated ARV1-FLAG protein and *vice versa*.

Both Anti-MYC and anti-FLAG antibodies co-immunoprecipitated ARV1-MYC and ARV1-FLAG (Fig. 11, *C* and *D*, mixed lysate) the negative control IgG antibodies did not pull down either protein from mixed lysates (Fig. 11, *C* and *D*). Interestingly, two proteins were pulled down with molecular weights of ∼55- and 25 to 30 kDa, with the 55 kDa protein being the predominant form.

**Figure 12:**
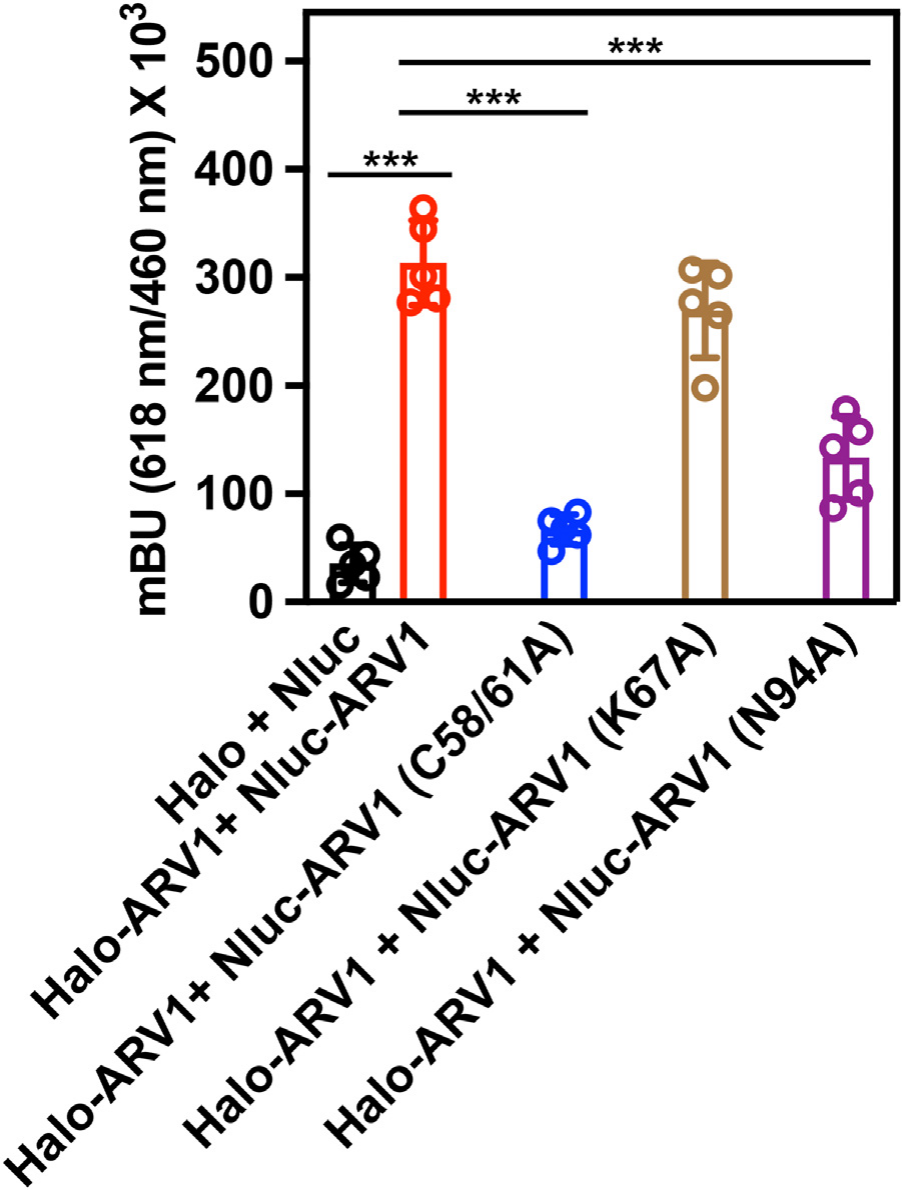
**Certain conserved amino acids are required for lipid binding.** The NanoBRET assay was performed with cells expressing either Halo-ARV1-Nluc-ARV1 (*red* bar), Halo-ARV1-Nluc-ARV1 (C58/C61A) (*blue* bar), Halo-ARV1-Nluc-ARV1 (K67A) (*brown* bar), or Halo-ARV1-Nluc-ARV1 (N94A) (*purple* bar) proteins. Halo-Nluc–expressing cells served as a negative control. Values are mean ± S.D. ∗∗∗*p* ≤ 0.0001. (n = 5). Two way ANOVA was used for statistical analysis.

Finally, we used the NanoBRET assay to test if there was a direct correlation between loss of lipid binding in our HTRF assay and loss of oligomerization (Fig. 12). In this case, we tested full-length ARV1 site-specific mutants.

Cells co-expressing full-length ARV1 had a robust BRET signal indicating the presence of a protein–protein interaction (Halo + Nluc *vs*. Halo-ARV1 + Nluc-ARV1; red bar). Recall the 6XHIS-ARV1-N98 mutant carrying C58/C61A mutations showed reduced binding affinity for PG (Table 2). Cells expressing a full-length ARV1 C58/61A mutant gave a very low BRET signal that was like that seen for the negative control (Halo-ARV1 + Nluc-ARV1 (C58/61A) (blue bar) *vs*. Halo + Nluc). While the 6XHIS-N98 harboring a K67A mutation showed reduced PG binding in our HTRF assay (Table 2), it gave a robust BRET signal when expressed in cells (Halo-ARV1 + Nluc-ARV1 (K67A) *vs*. Halo-ARV1 + Nluc-ARV1; brown bar), suggesting that K67 plays an important role in lipid binding but not in oligomerization. We also showed that the N94A mutant showed a reduction in PI(4)P binding (Table 2). Expressing this mutant in cells reduced, but did not eliminate, the BRET signal when compared to the negative control vectors (Halo-ARV1 + Nluc-ARV1 (N94A) *vs*. Halo-ARV1 + Nluc-ARV1; purple bar). Thus, there are at least two types of mutations affecting function, those that disrupt oligomerization and those that interfere with lipid binding.

## Discussion

If and how ARV1 regulates lipid transport has remained a mystery for over 25 years. *Arv1*^*−/−*^ KO mice fed a high fat diet do not accumulate cholesterol in their livers (42, 43). They do have elevated bile acid levels, thus the lack of accumulation of cholesterol may be due to its conversion to bile acids due to loss of proper intracellular transport out of the ER (43). Studies in yeast using mutants lacking ScArv1 have suggested ScArv1 functions in sterol transport (36, 66), as well as sphingolipid and GPI anchor biosynthesis (10, 34, 35, 67), as ceramides and GPI intermediates accumulate in these cells (10, 35, 67, 68). The catalytic step where Arv1 is proposed to function in the synthetic pathway for GPI anchor biosynthesis requires a lipid flippase activity (34) and is conserved in humans (69) and *Trypanosoma brucei* (70). While these studies reinforce the importance of ARV1 in lipid transport and/or biosynthesis, to date, no mechanistic studies have been performed attempting to uncover its exact molecular function(s). Here, our efforts focused on determining if ARV1 possessed lipid-binding activity.

While earlier cell-based proteomic studies suggested that human ARV1 possessed cholesterol-binding activity, definitive proof has been lacking using purified protein and *in vitro* biochemical assays. Here, we showed for the first time that recombinant human ARV1 bound cholesterol using liposome-binding assays. The liposome-binding assay is a highly validated method for determining lipid–protein interactions. Various liposome assays have been used to determine lipid binding specificity for cytochrome c and model its insertion into phosphatidic acid liposomes (71), the binding to and extraction of cholesterol by ORP9 (72), and the bilayer lipid transfer of cholesterol by NPC1 (73). Cholesterol is a planar, rigid, and hydrophobic molecule that has a neutral charge (74). Thus, the binding of ARV1 to cholesterol is most likely direct and independent of electrostatic interactions.

ARV1 also bound PLs and PIPs. Many families of lipid transporters bind and transport/transfer multiple lipid species (30). Several lipid scramblases transfer phospholipids like PS, PC, and PE (28). Phospholipid flippases also transfer PS and PE between PM bilayers (28). Human OSBP and the yeast ortholog, Osh4, bind cholesterol and PI(4)P and exchange these lipids between ER and Golgi membranes (21). Interestingly, inhibition of OSBP results in the accumulation of cholesterol and lipid droplets in the ER, with a concomitant reduction in the trans-Golgi (75). Human ORP5/8 proteins transfer PS to the PM in exchange for removal of PI(4)P, which regulates PIP signaling through regulating PI(4,5)P_2_ levels (1). Several LTPs have been shown to possess a second function that is tied to lipid transfer (76).

ARV1 bound sulfatide, which makes up the major sulfoglycolipid in the nervous system (76). There is a step in the synthesis of sulfatide that produces GalCer by adding UDP onto ceramide (76). A sulfate group is then added to form sulfatide. *ScArv1Δ* cells accumulate multiple ceramide species in the ER (34, 35). Based on this information, questions that could be asked include, do ceramides accumulate in the brains of individuals carrying *hARV1* mutations and does this lead to reduced sulfatide levels and increased myelin defects (76) and is there a connection between lack of human ARV1 binding to sulfatide and the neurological defects seen in *hARV1* variant patients (77, 78, 79)? We point out that Annexin A2 also bound sulfatide, which represents a possible new lipid-binding function for this protein.

Funato et al. (34) have shown that ScArv1 can localize within small cellular foci that may be oligomers of ScArv1 that are localized at membrane contact sites (80, 81, 82). Our evidence strongly suggests that ARV1 needs to dimerize for it to bind lipids. Sucrose gradients revealed an ARV1 protein of ∼55 kDa, which would constitute a dimer, and co-immunoprecipitation further validated these results. Being localized at contact sites would facilitate ARV1-dependent lipid transfer/transport activity.

**Figure 13:**
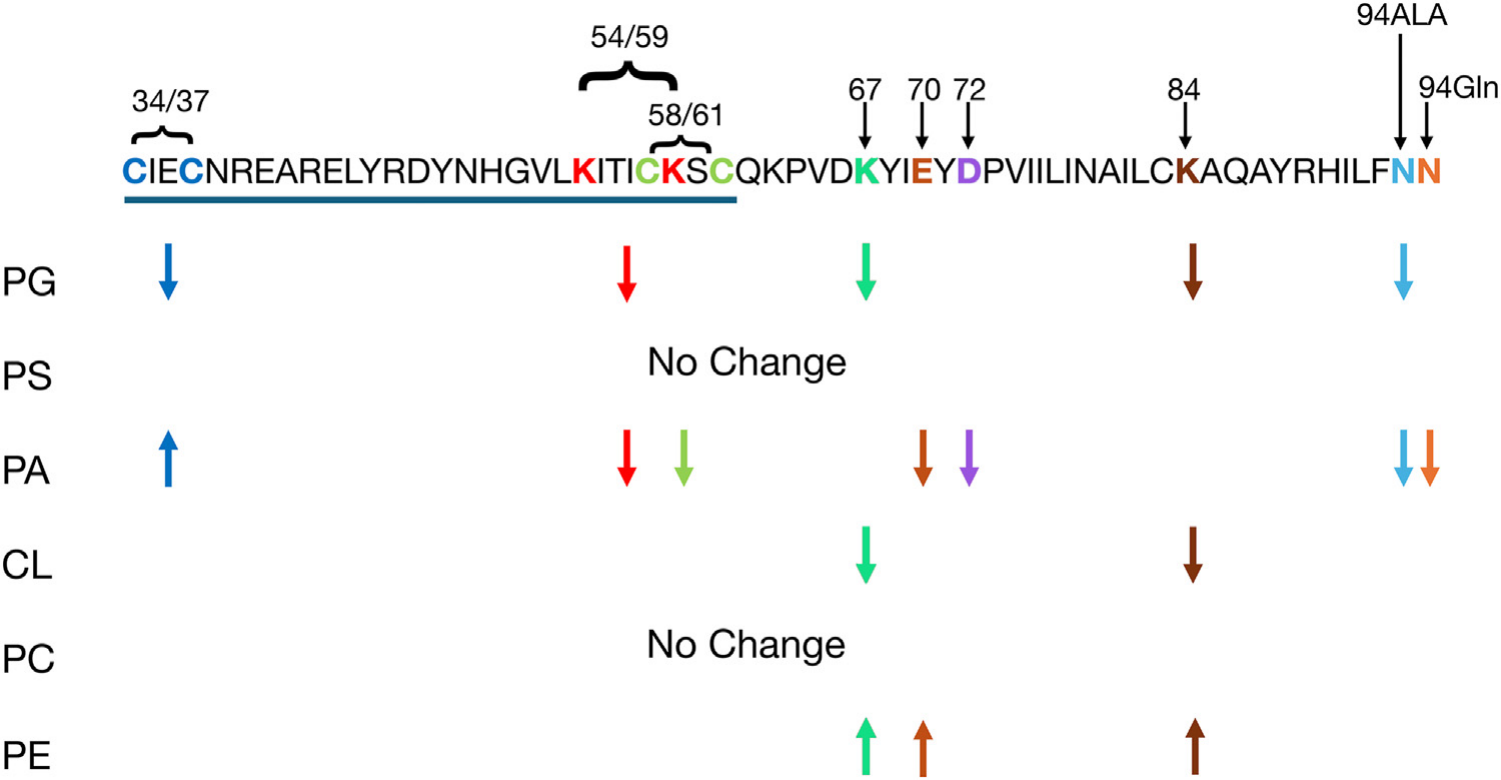
**Schematic diagram indicating the effects of various amino acid mutations on lipid binding.** Schematic of the AHD of human ARV1. The zinc-binding domain is underlined. Conserved amino acids mutated are color coded with unique colors. In the case of double mutations, the color coding is the same. The arrows are the same color as the amino acids and represent whether lipid binding was increased (*up arrow*) or decreased (*down arrow*).

Our truncation analysis and site-directed mutagenesis studies revealed the importance of the zinc-binding motif and cysteine clusters for lipid-binding (Fig. 13). All lipid-binding activity of ARV1 resided within the AHD domain. Lipid binding occurred between amino acids 30 and 97 of the AHD. An intact zinc-binding motif was necessary for lipid binding and the cysteine clusters were critical for binding specificity. The C34/C37A mutant showed enhanced PA binding over the N98 protein, while the C58/C61A mutant showed reduced PA binding. The K54/K59A mutant also showed decreased PA-binding affinity as well as binding to PG. This may indicate that the amino acid cluster, 54-KITICKSC-61, plays an important role in defining lipid-binding specificity. Mutations within another amino acid cluster, 67-KYIEYD-72, also decreased PA binding but increased PE binding, suggesting this cluster may drive PL species preference.

None of the mutations affected PS binding. This is interesting since the AHD contains a weak consensus PS-binding site (83, 84) and we did mutate K54/K59, and K67, within this site. ARV1 binding to cholesterol may be through the single CRAC site found within the AHD (44-LYRDYNHGVLK-54) (85). ARV1 contains several CRAC sites throughout the entire protein including within potential transmembrane domains.

We performed a motif search (https://www.genome.jp/tools/motif/) using human ARV1 that uncovered Pfam domains that included 1) a cysteine-rich ADD domain (ATRX-Dnmt3-Dmnt3l, Ad-DATRX) (32-YRCIECNQEAKELYRDYNHGVLKITICKSCQK-64) found in ATRX proteins, 2) a domain of unknown function that is found in a family of halobacteria (DUF7573) (21-ATPTAASASCQYRCIECNQEAKELYRDYNHGVLKITICKSCQ-63), and 3) the AHD domain. Mutations in the human ATRX histone-associated protein have been linked to a severe form of X-linked mental retardation called ATR-X syndrome (86). Interestingly, an *ARV1* variant harboring a Cys34 to Tyr mutation, which is within this ADD domain, has been linked to epileptic encephalitis and cognitive disorders. The human protein also contains a weak SH2 domain (66-DKYIEYD-72). SH2 domains bind PM phospholipids that are involved in mitogenic receptor signaling (87). Mutating K67 and D72 within this domain resulted in changes in PL preference and affinity.

ARV1-N98 bound many PLs having a negative charge, so we believe electrostatic charge differences do play a role in PL binding, but we also believe that other physical interactions are involved. While ARV1-N98 did bind several PLs with a negative charge (PA, PS, PG, CL) and not those with a neutral charge (PC, PE), it did not bind PI, PI(4,5)P_2_, or PI(3,5)P_2_ that all have a negative charge. On the other hand, ARV1-N98 bound the monophosphorylated PIPs, PI(3)P, PI(4)P, and PI(5)P, which all carry a negative charge.

We have found that human ARV1 can be phosphorylated *in vitro* on Tyr68 within the SH2 domain by the epidermal growth factor receptor (EGFR) and colocalizes with this receptor in HEK293 cells (*K.K. Frietze and J.T. Nickels, Jr., unpublished results*). EGFR signaling has been linked to hepatic stellate cell activation during MASH (88) and adipose tissue activation during obesity (89). PKC activity, which is activated by EGFR-dependent hydrolysis of PI(3,4,5)P_3_ and generation of diacylglyceride (90), has been linked to obesity (91), T2D (92), and MASH (93). If ARV1 transports PI(4)P to the PM, possibly at ER-PM contact sites, triggered by EGFR-dependent phosphorylation on Tyr68, this could supply a precursor for PI(3,4,5)P_3_ synthesis. Whether EGFR phosphorylation of ARV1 regulates lipid binding and/or transport during mitogenic signaling requires further *in vitro* and cell-based studies.

## Experimental procedures

### Materials

Lipid overlay membrane strips and arrays were purchased from Echelon Biosciences. Site-directed mutagenesis was carried out using the QuikChange Site-Directed Mutagenesis kit (Agilent, #200518). Unless otherwise indicated, all chemicals and reagents were purchased from Thermo Fisher Scientific. Lipids were purchased from Avanti Polar Lipids and Millipore Sigma either lyophilized or dissolved in chloroform at a concentration of 10 mg/ml. Biotinylated PIPs were purchased from Echelon Biosciences.

### Protein expression in *E*. *coli*

Plasmid pGEX-4T1 (Millipore Sigma #GE28-9545-49) was used to express GST-tagged proteins. Plasmid pET-28a(+) (GenScript) was used to express N-terminal 6XHIS-tagged protein. All plasmids are listed in Table 4. Schematic diagrams of various constructs generated are shown in Fig. S1. BL21 cells were grown to an *A*_*600*_ ∼0.5 in LB medium containing 100 μg/ml kanamycin. Fusion-tagged protein expression was induced by 0.5 mM IPTG (Millipore Sigma, #I6758-1G). Cells were pelleted and stored at −80 °C.

**Table 4:** Plasmids used in this study

Plasmids	Construction	Source or reference
pGEX-4T1	Plasmid expressing an amino-terminal GST-tagged fusion protein	Millipore, Sigma
Derivatives		
pGEX-4T1-GST-ARV1	Expresses full length ARV1	This study
pGEX-4T1-GST-N98	Expresses amino-terminal 98 amino acids of ARV1	This study
pGEX-4T1-GST-N90	Expresses amino-terminal 90 amino acids of ARV1	This study
pGEX-4T1-GST-N70	Expresses amino-terminal 70 amino acids of ARV1	This study
pGEX-4T1-GST-N60	Expresses amino-terminal 60 amino acids of ARV1	This study
pGEX-4T1-GST-N50	Expresses amino-terminal 50 amino acids of ARV1	This study
pGEX-4T1-GST-N30	Expresses amino-terminal 30 amino acids of ARV1	This study
pGEX-4T1-GST-N10	Expresses amino-terminal 10 amino acids of ARV1	This study
pGEX-4T1-N98	Expresses amino-terminal 98 amino acids of ARV1	This study
Derivatives		
pGEX-4T1-GST-N98 (C34/37A)	GST-N98 harboring Cys34Ala/Cys37/Ala mutations	This study
pGEX-4T1-GST-N98 (K54/59A)	GST-N98 harboring Lys54Ala/Lys59/Ala mutations	This study
pGEX-4T1-GST-N98 (C58/61A)	GST-N98 harboring Cys58Ala/Cys61/Ala mutations	This study
pGEX-4T1-GST-N98 (K67A)	GST-N98 harboring Lys67/Ala mutation	This study
pGEX-4T1-GST-N98 (E70A)	GST-N98 harboring Glu70/Ala mutation	This study
pGEX-4T1-GST-N98 (K84A)	GST-N98 harboring Lys84/Ala mutation	This study
pGEX-4T1-GST-N98 (N94A)	GST-N98 harboring Asn94/Ala mutations	This study
pGEX-4T1-GST-N98 (N94Q)	GST-N98 harboring Asn94/Gln mutation	This study
pET-28a(+)	Plasmid expressing an amino-terminal 6XHIS -tagged fusion protein	GenScript, Piscataway, NJ
Derivatives		
pET-28a(+)-6XHIS-ARV1	Expressing full-length ARV1	This study
pET-28a(+)-6XHIS-N98	Expressing amino-terminal 98 amino acids of ARV1	This study
pET-28a(+)-6XHIS-N98	Plasmid expressing an amino-terminal 6XHIS-N98 fusion protein	This study
Derivatives		
pET-28a(+)-6XHIS-N98 (C34/37A)	6XHIS-N98 harboring Cys34Ala/Cys37/Ala mutations	This study
pET-28a(+)-6XHIS-N98 (K54/59A)	6XHIS-N98 harboring Lys54Ala/Lys59/Ala mutations	This study
pET-28a(+)6XHIS-N98 (C58/61A)	6XHIS-N98 harboring Cys58Ala/Cys61/Ala mutations	This study
pET-28a(+)-6XHIS-N98 (K67A)	6XHIS-N98 harboring Lys67/Ala mutation	This study
pET-28a(+)-6HIS-N98 (E70A)	6XHIS-N98 harboring Glu70/Ala mutation	This study
pET-28a(+)-6XHIS-N98 (D72A)	6XHIS-N98 harboring Asp72/Ala mutation	This study
pET-28a(+)-6XHIS-N98 (K84A)	6XHIS-N98 harboring Lys84/Ala mutation	This study
pET-28a(+)-6XHIS-N98 (N94A)	6XHIS-N98 harboring Lys84/Ala mutation	This study
pET-28a(+)-6XHIS-N98 (N94Q)	6XHIS-N98 harboring Asn94/Ala mutations	This study
	6XHIS-N98 harboring Asn94/Gln mutation	This study

### Protein purification

Cell pellets were resuspended in lysis buffer (50 mM sodium phosphate buffer, pH 7.2, containing 300 mM NaCl, 0.1% Triton X-100, and 30 mM imidazole) containing an EDTA-free protease inhibitor cocktail tablet (Millipore Sigma, #4693132001). Hundred microliters of 200 mg/ml of lysozyme (Millipore Sigma, #L6876) was added to cell pellet lysates on ice. Six microliters of benzonase (Millipore Sigma, #9025-65-4) was then added and lysates were mechanically agitated until a clear solution was observed. Cells were spun down at 12,000*g* for 10 min at 4 °C and the clear supernatant was used for purification.

N-terminal GST-tagged fusion proteins were affinity purified using GST-coated beads (Millipore Sigma, #G4510). Briefly, proteins were affinity immunoprecipitated from cell lysates using lysis buffer (50 mM sodium phosphate buffer, pH 7.2, containing 300 mM NaCl, 0.1% Triton X-100) by incubating cell supernatants with 300 μl of a GST-coated bead slurry and rotated for 2 h at 4 °C. Beads were spun down for 5 min at 4000*g* at 4 °C and washed three times with ice cold PBS. Elution was performed by using 10 mM glutathione diluted in 500 μl of 50 mM Tris–HCL buffer, pH 8.8, containing 0.1% Triton X-100, and vortexing for 5 min on ice. GST protein was purified and used as a negative nonspecific binding control.

N-terminal 6XHIS-tagged fusion proteins were affinity purified using TALON superflow metal affinity resin (Clontech #635506). Briefly, lysates were run through the column twice before washing. Resin columns were washed with (1) 30 ml of 50 mM sodium phosphate buffer, pH7.2, containing 300 mM NaCl and 30 mM imidazole, (2) 30 ml of 20 mM sodium phosphate buffer, pH7.2, containing 500 mM NaCl and 30 mM imidazole, (3) 30 ml of 20 mM sodium phosphate buffer, pH7.2, containing 150 mM NaCl and 30 mM imidazole, and (4) 30 ml of 20 mM sodium phosphate buffer, pH7.2, containing 150 mM NaCl and 150 mM imidazole. Tagged-proteins were eluted in step-wise fashion as 5 μl fractions using 20 mM sodium phosphate, pH 7.2, containing 150 mM NaCl, 500 mM imidazole, 0.1% Triton X-100, and 5% glycerol. Eluted proteins were stored at −80 °C.

### Western analysis

Cell lysate protein levels were determined using the Pierce BCA protein assay kit (Thermo Fisher Scientific #A55860). Twenty five micrograms of protein was loaded on gels. Proteins were resolved by SDS-PAGE using 4 to 20% polyacrylamide gels and subsequently transferred to 0.2 μm nitrocellulose membranes. Membranes were blocked with TBST containing 2% nonfat milk for 60 min at room temperature then washed three separate times with TBST containing 2% nonfat milk for 30 min at room temperature. An anti-6XHIS monoclonal antibody (R&D Systems #MAB050, Minneapolis, MN) at a 1:1000 dilution or an anti-GST monoclonal antibody (Millipore Sigma #G1160) at a 1:1500 dilution were incubated with the corresponding membranes for 1 h at room temperature. Membranes were then washed 8 times for 30 min with TBST containing 2% nonfat milk. A final wash using TBST alone was performed prior to the addition of secondary antibodies. Membranes were visualized using Immobilon Western Chemiluminescent horseradish peroxidase substrate (Millipore catalog #WBKLS0500) and the GE ImageQuant LAS 3000 imager.

### Lipid overlay far westerns

Lipid overlays were blocked in TBST-BSA (100 mM Tris–HCl buffer, pH 7.5, 150 mM NaCl, 0.5% Tween 20, 3% bovine serum albumin) for 1 h and subsequently washed eight separate times with TBST for 2 min each. Overlays were incubated with 0.5 μg/ml GST-tagged or 6XHIS-tagged proteins in 3% TBST-BSA buffer containing 0.1% Triton X-100, for 1 h at room temperature and washed as above. Overlays were incubated with either anti-GST or anti-6XHIS monoclonal antibodies at a 1:1000 dilution in TBST-BSA for 1 h at room temperature followed by eight washes in TBST. Overlays were visualized using Immobilon Western Chemiluminescent horseradish peroxidase substrate and protein–lipid interaction was detected using a GE ImageQuant LAS 3000 imager.

### PC:PL liposome preparations

Lyophilized lipids were reconstituted in chloroform at 10 mg/ml and stored in glass tubes with Teflon tops at −20 °C until use. One milliliter of 10 mg/ml stock was dried under a stream of nitrogen in a glass tube until a lipid cake/film was formed. The lipid cake was then rehydrated in BLT rehydration buffer (5 mM Tris/HCl, pH 7.4, containing 150 mM NaCl) to a concentration of 1 mg/ml. Lipid suspensions were subjected to three freeze/thaw cycles using a dry ice ethanol/-50 °C water bath to encapsulate the buffer and improve liposome formation. The suspension and lipid extruder were equilibrated to temperature for 10 min and liposomes were extruded using 0.1 μM polycarbonate membranes (Avestin). Liposomes were made by mixing stock lipid PL (1 mg/ml) with PC (10 mg/ml).

Cholesterol composition liposomes were rehydrated with cholesterol rehydration buffer (10 mM Hepes, pH 7.4, containing 150 mM KCl).

Sulfatide liposomes were generated as previously described (94). All lipids were resuspended in organic solvents as per manufacturer’s instructions. Liposomes were prepared with mixing PC:PE:cholesterol:sulfatides (1:1:1:4) and subsequently dried down by lyophilization to obtain lipid films. Lipids were hydrated in 20 mM Tris–HCL, pH 6.8, containing 100 mM NaCl at a concentration of 1 mg/ml.

PI(4)P liposomes were generated as described by Knodler *et al.* (95), with some modifications. Briefly, liposomes were generated at a lipid concentration of 10 mg/ml. Liposomes contained PC (60%), PI (30%), PE (8%), and PI(4)P (2%). Lipid solution was dried down and resuspended in liposome buffer (20 mM Hepes–HCL, pH 7.4, containing 1 mM EDTA and 50 mM potassium acetate). Liposomes were formed by sonication on ice.

### Protein-lipid liposome binding assay

Liposomes were serial diluted in floatation buffer (100 mM NaCl, 2 mM EDTA, pH 5.5) to a final concentration of 0.1 mg/ml. Nine hundred microliters of liposome solution was placed in clear ultracentrifuge tubes and mixed with 100 μl of 0.01 μg/ml of protein. The liposome/protein solution was incubated at room temperature for 1 h with occasional shaking. An initial 100 μl of liposome/protein solution (liposomes) was removed and the remaining 900 μl was centrifuged at 50,000*g* for 20 min at 4 °C. The pellet was resuspended in 800 μl of fresh floatation buffer, centrifuged as above, and 800 μl of supernatant was removed (wash 1). This was repeated a second time and again 800 μl of supernatant was removed (wash 2). The final pellet was resuspended in 100 μl of floatation buffer plus SDS treatment buffer (pellet). Thirty microliters of each fraction was run on a 15% SDS-PAGE gel and probed with an anti-GST monoclonal antibody at a 1:1500 dilution.

### Sucrose density gradient centrifugation

Sucrose density gradients of 2.5 to 25% were made in PBS. Gradients were spun at 132,000*g* for 19 h. Fractions were obtained from the top of the tubes and used for protein detection by SDS-PAGE and western analysis.

### Yeast two-hybrid assay

Yeast cells harboring p-BD or p-AD plasmids expressing full-length or ARV1-N98 fusion proteins were grown in selective medium to maintain plasmid selection. Serial dilutions were then dropped onto protein–protein interaction selective medium. Plates were incubated at 37 °C, and growth was determined after 7 days.

### NanoBRET cell-based assay

The Promega NanoBRET cell-based protein–protein interaction detection system (Promega, #1662) was used for detecting ARV1 interactions. NanoBRET system detection is based on energy transfer between an energy donor NanoLuc-fusion protein (blue-shifted) and an energy acceptor HaloTag-fusion protein (red-shifted) (96). The degree of binding is calculated by determining the NanoBret signal ratio. ARV1 and ARV1-N98 proteins were cloned into the supplied vectors as N-terminal–tagged fusion proteins.

HEK293T cells that were grown overnight to ∼80 to 90% confluency were resuspended in fresh phenol red–free Dulbecco’s modified Eagle’s medium (DMEM) + 10% FBS. A DNA transfection reagent containing 1 μg of NanoLuc-fusion vector + 2 μg HaloTag-fusion vector, Enhancer, and Effectene buffer was incubated for 2 min. Effectene was subsequently added, and the solution was incubated for an additional 10 min before being added to cells. Once added, cells were left to incubate overnight in a tissue culture incubator. Cells were then washed in clear DMEM minus FBS and resuspend in clear DMEM + 5% FBS. Fifteen microliters containing 20,000 cells were pipetted into individual wells of a 384-well plate, and 2 μl of 1:160 dilution of NanoBRET ligand (in FBS-free clear DMEM) was then added to each well, after which the plate was spun at 1000 rpm for 30 s. The plate was incubated at 37 °C in a tissue culture incubator for 6 h. Two microliters of a 1:160 dilution of NanoBRET substrate was added and plates were read immediately using a PerkinElmer EnVision Xcite Multilabel plate reader.

### HTRF phospholipid lipid-binding assay

An HTRF assay was developed for 6XHIS-ARV1-N98-lipid binding. HTRF measures analyte interactions through fluorescence resonance energy transfer technology and time-resolved measurements (53). The interaction between two molecules can be quantified through binding each individual molecule involved with a fluorescent label and then analyzing the transfer of energy between the initial donor fluorophore and the secondary acceptor fluorophore. Following excitation of the first fluorophore, transfer of energy can only occur if the molecules bound with the fluorescent tags are within proximity to each other.

Assays were plated in 384-well flat bottom microplates (Greiner Bio, catalog #784075-25). Proteins were diluted using binding buffer (20 mM Hepes buffer, pH 7.2, containing 0.1% BSA, and 0.01% Tween20) to a stock concentration of 0.75 μM, and biotinylated phospholipids were dispensed, dried under nitrogen gas, and resuspended in binding buffer to a stock concentration of 50 μM. The lipid stock solution was diluted by 1:2 for a series of 12 serial dilutions. For the assay, in sequential order, 6 μl of binding buffer was loaded into the plate in triplicate for each dilution of phospholipid. To this, 2 μl of 0.75 μM 6XHIS-ARV1-N98 was added, followed by 2 μl of biotinylated phospholipid. Immediately following each addition loaded into the plate, the plate was centrifuged for 1 min at 1000 rpm to bring the contents to the bottom of the well. Next, detection antibodies were mixed with detection buffer (20 mM Hepes buffer, pH 8.5, containing 0.01% BSA and 200 mM KF). Antibodies were a MAb anti-6XHIS Europium kryptate-conjugated D1 fluorescent antibody (Cisbio, #61HISKLA) and Streptavidin-conjugated d2 fluorescent antibody (Cisbio, #610SADLA). For each 1000 μl of detection buffer, 5 μl of each antibody was added just prior to loading 10 μl into each well. The plate was again centrifuged for 1 min at 1000 rpm and stored in a sealed container overnight, in the dark. Plates were read on a PerkinElmer EnVision Xcite Multilabel plate reader.

### HTRF phospholipid competition assays

The competition assays followed a similar protocol as described for the HTRF described above. In the competition assay, both protein and biotinylated lipid were plated at a constant concentration, and the competing nonbiotinylated lipid was loaded at increasing concentrations. Proteins and biotinylated phospholipids were diluted with binding buffer and plated to a constant concentration. Protein concentration was 0.75 μM while the concentration of the biotinylated phospholipid was used at a concentration that gave an 80% saturated binding signal. Competitor lipid was diluted to a stock concentration of 50X the loading concentration of the tagged lipid and diluted further for twelve 1:2 serial dilutions.

The assay was as follows and added in sequential order using 384-well plates. Four microliters of binding buffer was added, followed by 2 μl of protein, followed by 2 μl of biotinylated lipid. The plate was centrifuged for 1 min at 1000*g*, after each addition. The detection buffer containing the MAb anti-6XHIS Europium kryptate-conjugated D1 fluorescent antibody and Streptavidin-conjugated d2 fluorescent antibody was added to each well, followed by a final spin before overnight incubation in the dark. Plates were read using PerkinElmer EnVision Xcite Multilabel plate reader.

### HTRF statistical analysis

HTRF data was analyzed and graphed using GraphPad Prism 10 software. Curves were generated with three points per concentration point, per replicate and graphed as mean ± S.D.

Curves were logarithmically transformed and normalized to a common scale before being fit with a nonlinear regression of the agonist against the normalized response. This generated half maximal effective concentration values (EC_50_; M) that could then be compared between different lipids. In the context of our study, EC_50_ values were defined as a measure of protein sensitivity to binding to a particular lipid within a dose response curve. For phospholipid competition analysis, IC_50_; M values were determined using nonlinear regression of the inhibitor against normalized response.

The EC_50_ and IC_50_ values for replicate experiments per lipid–protein combination were recorded and graphed to compare their distributions between replicates to determine reproducibility and between lipid–protein combinations to determine differences in apparent affinity of binding. A one-way ANOVA was performed to first identify if there were differences in the distributions of EC_50_ values. When significant differences were calculated, a student’s 2-sample *t* test was used to establish if independent curves were statistically different from one another.

Statistical analysis of EC_50_ distributions showed significant differences between binding curves of the specific lipids using the ARV1-N98 protein (ANOVA *p* value < 0.0001). We next determined how different EC_50_ values of individual phospholipids differed from PG. A *t* test determined each curve was statistically different (*p* < 0.01). We determined that there were no statistical differences comparing PS to CL EC_50_ values and PC to PE EC_50_ values.

## Data availability

All data is contained within the manuscript.

## Supporting information

This article contains supporting Information.

## Conflict of interest

All authors are present or were past employees of Genesis Global Group, Inc.

## Supporting information

Supplementary figure **Schematic diagram of various expressed tagged fusion proteins.** The protein structure of full-length ARV1, ARV1-N98, and various ARV1 truncations are shown. Amino acids that were mutated are indicated (C, cysteine; K, lysine; E, glutamate; N, asparagine; D, aspartate; Q, glutamine). AHD, ARV1 homology domain; TM, transmembrane domain; ZBM, zinc-binding motif.
