# Supplementary material for: The sterol-regulating human ARV1 binds cholesterol and phospholipids through its conserved ARV1 homology domain
Source: J Biol Chem. 2025 Feb 12;301(3):108306. doi: 10.1016/j.jbc.2025.108306 (PMC11952846; doi:10.1016/j.jbc.2025.108306)
Supplement: Supplementary figure — Schematic diagram of various expressed tagged fusion proteins. The protein structure of full-length ARV1, ARV1-N98, and various ARV1 truncations are shown. Amino acids that were mutated are indicated (C, cysteine; K, lysine; E, glutamate; N, asparagine; D, aspartate; Q, glutamine). AHD, ARV1 homology domain; TM, transmembrane domain; ZBM, zinc-binding motif. [file mmc1.pdf]

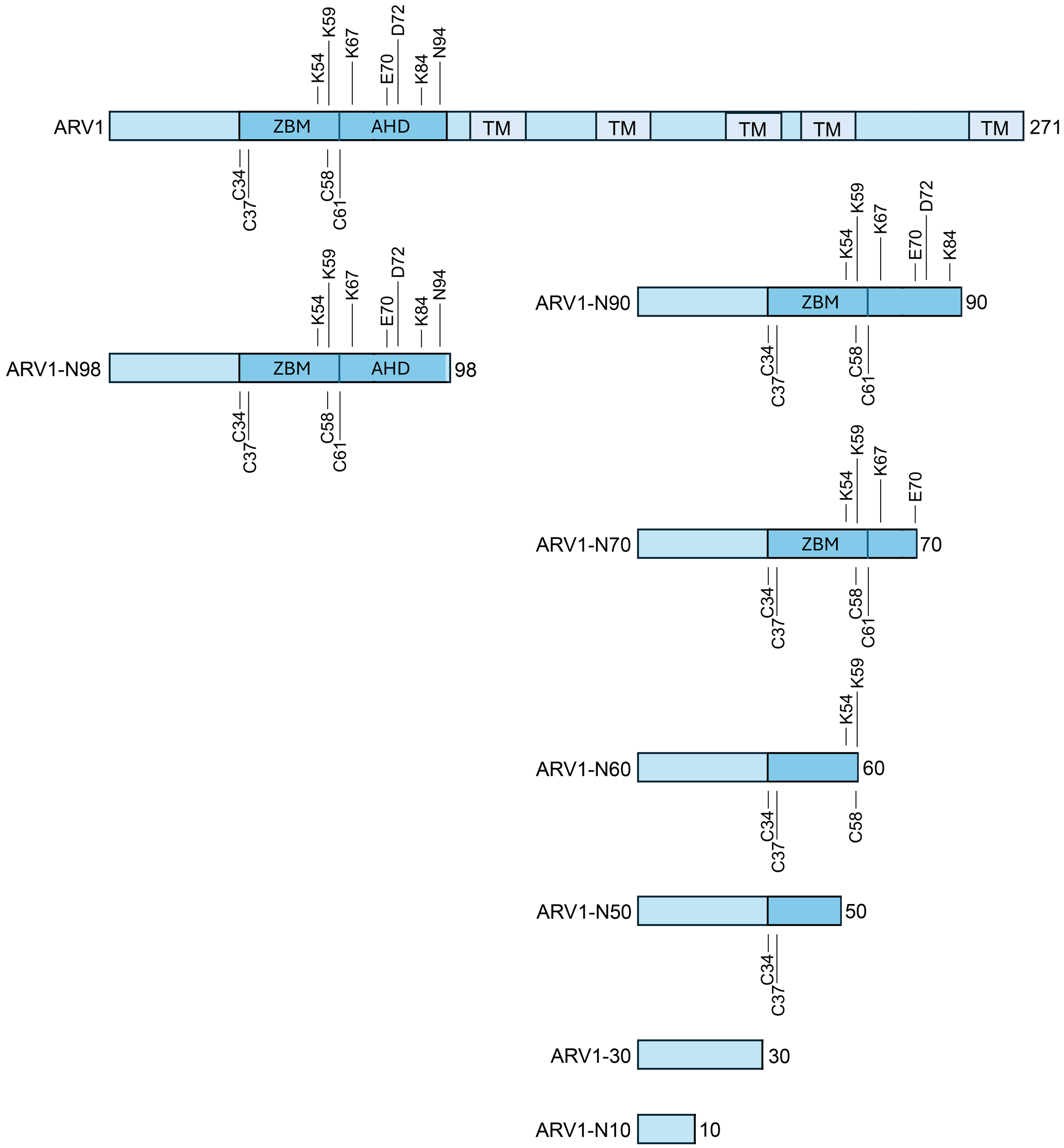

ARV1
ZBM
AHD
TM
TM
TM
TM
TM
271
K54
K59
K67
E70
D72
K84
N94
C34
C37
C58
C61
ARV1-N98
98
ARV1-N90
90
ARV1-N70
70
ARV1-N60
60
ARV1-N50
50
ARV1-30
30
ARV1-N10
10

Fig. S1. Schematic diagram of various expressed tagged fusion proteins. The protein structure of full-length ARV1, ARV1-N98, and various ARV1 truncations are shown. Amino acids that were mutated are indicated (C, cysteine; K, lysine; E, glutamate; N, asparagine; D, aspartate; Q, glutamine). AHD, ARV1 homology domain; ZBM, zinc binding motif; TM, transmembrane domain.
